# Measurement of $\tau$ polarisation in $Z/\gamma ^{*}\rightarrow \tau \tau$ decays in proton–proton collisions at $\sqrt{s}=8$ TeV with the ATLAS detector

**DOI:** 10.1140/epjc/s10052-018-5619-1

**Published:** 2018-02-24

**Authors:** M. Aaboud, G. Aad, B. Abbott, O. Abdinov, B. Abeloos, S. H. Abidi, O. S. AbouZeid, N. L. Abraham, H. Abramowicz, H. Abreu, R. Abreu, Y. Abulaiti, B. S. Acharya, S. Adachi, L. Adamczyk, J. Adelman, M. Adersberger, T. Adye, A. A. Affolder, Y. Afik, T. Agatonovic-Jovin, C. Agheorghiesei, J. A. Aguilar-Saavedra, S. P. Ahlen, F. Ahmadov, G. Aielli, S. Akatsuka, H. Akerstedt, T. P. A. Åkesson, E. Akilli, A. V. Akimov, G. L. Alberghi, J. Albert, P. Albicocco, M. J. Alconada Verzini, S. C. Alderweireldt, M. Aleksa, I. N. Aleksandrov, C. Alexa, G. Alexander, T. Alexopoulos, M. Alhroob, B. Ali, M. Aliev, G. Alimonti, J. Alison, S. P. Alkire, B. M. M. Allbrooke, B. W. Allen, P. P. Allport, A. Aloisio, A. Alonso, F. Alonso, C. Alpigiani, A. A. Alshehri, M. I. Alstaty, B. Alvarez Gonzalez, D. Álvarez Piqueras, M. G. Alviggi, B. T. Amadio, Y. Amaral Coutinho, C. Amelung, D. Amidei, S. P. Amor Dos Santos, S. Amoroso, G. Amundsen, C. Anastopoulos, L. S. Ancu, N. Andari, T. Andeen, C. F. Anders, J. K. Anders, K. J. Anderson, A. Andreazza, V. Andrei, S. Angelidakis, I. Angelozzi, A. Angerami, A. V. Anisenkov, N. Anjos, A. Annovi, C. Antel, M. Antonelli, A. Antonov, D. J. Antrim, F. Anulli, M. Aoki, L. Aperio Bella, G. Arabidze, Y. Arai, J. P. Araque, V. Araujo Ferraz, A. T. H. Arce, R. E. Ardell, F. A. Arduh, J.-F. Arguin, S. Argyropoulos, M. Arik, A. J. Armbruster, L. J. Armitage, O. Arnaez, H. Arnold, M. Arratia, O. Arslan, A. Artamonov, G. Artoni, S. Artz, S. Asai, N. Asbah, A. Ashkenazi, L. Asquith, K. Assamagan, R. Astalos, M. Atkinson, N. B. Atlay, K. Augsten, G. Avolio, B. Axen, M. K. Ayoub, G. Azuelos, A. E. Baas, M. J. Baca, H. Bachacou, K. Bachas, M. Backes, P. Bagnaia, M. Bahmani, H. Bahrasemani, J. T. Baines, M. Bajic, O. K. Baker, P. J. Bakker, E. M. Baldin, P. Balek, F. Balli, W. K. Balunas, E. Banas, A. Bandyopadhyay, Sw. Banerjee, A. A. E. Bannoura, L. Barak, E. L. Barberio, D. Barberis, M. Barbero, T. Barillari, M.-S. Barisits, J. T. Barkeloo, T. Barklow, N. Barlow, S. L. Barnes, B. M. Barnett, R. M. Barnett, Z. Barnovska-Blenessy, A. Baroncelli, G. Barone, A. J. Barr, L. Barranco Navarro, F. Barreiro, J. Barreiro Guimarães da Costa, R. Bartoldus, A. E. Barton, P. Bartos, A. Basalaev, A. Bassalat, R. L. Bates, S. J. Batista, J. R. Batley, M. Battaglia, M. Bauce, F. Bauer, H. S. Bawa, J. B. Beacham, M. D. Beattie, T. Beau, P. H. Beauchemin, P. Bechtle, H. P. Beck, H. C. Beck, K. Becker, M. Becker, C. Becot, A. J. Beddall, A. Beddall, V. A. Bednyakov, M. Bedognetti, C. P. Bee, T. A. Beermann, M. Begalli, M. Begel, J. K. Behr, A. S. Bell, G. Bella, L. Bellagamba, A. Bellerive, M. Bellomo, K. Belotskiy, O. Beltramello, N. L. Belyaev, O. Benary, D. Benchekroun, M. Bender, N. Benekos, Y. Benhammou, E. Benhar Noccioli, J. Benitez, D. P. Benjamin, M. Benoit, J. R. Bensinger, S. Bentvelsen, L. Beresford, M. Beretta, D. Berge, E. Bergeaas Kuutmann, N. Berger, J. Beringer, S. Berlendis, N. R. Bernard, G. Bernardi, C. Bernius, F. U. Bernlochner, T. Berry, P. Berta, C. Bertella, G. Bertoli, I. A. Bertram, C. Bertsche, D. Bertsche, G. J. Besjes, O. Bessidskaia Bylund, M. Bessner, N. Besson, A. Bethani, S. Bethke, A. Betti, A. J. Bevan, J. Beyer, R. M. Bianchi, O. Biebel, D. Biedermann, R. Bielski, K. Bierwagen, N. V. Biesuz, M. Biglietti, T. R. V. Billoud, H. Bilokon, M. Bindi, A. Bingul, C. Bini, S. Biondi, T. Bisanz, C. Bittrich, D. M. Bjergaard, J. E. Black, K. M. Black, R. E. Blair, T. Blazek, I. Bloch, C. Blocker, A. Blue, U. Blumenschein, S. Blunier, G. J. Bobbink, V. S. Bobrovnikov, S. S. Bocchetta, A. Bocci, C. Bock, M. Boehler, D. Boerner, D. Bogavac, A. G. Bogdanchikov, C. Bohm, V. Boisvert, P. Bokan, T. Bold, A. S. Boldyrev, A. E. Bolz, M. Bomben, M. Bona, M. Boonekamp, A. Borisov, G. Borissov, J. Bortfeldt, D. Bortoletto, V. Bortolotto, D. Boscherini, M. Bosman, J. D. Bossio Sola, J. Boudreau, E. V. Bouhova-Thacker, D. Boumediene, C. Bourdarios, S. K. Boutle, A. Boveia, J. Boyd, I. R. Boyko, A. J. Bozson, J. Bracinik, A. Brandt, G. Brandt, O. Brandt, F. Braren, U. Bratzler, B. Brau, J. E. Brau, W. D. Breaden Madden, K. Brendlinger, A. J. Brennan, L. Brenner, R. Brenner, S. Bressler, D. L. Briglin, T. M. Bristow, D. Britton, D. Britzger, F. M. Brochu, I. Brock, R. Brock, G. Brooijmans, T. Brooks, W. K. Brooks, J. Brosamer, E. Brost, J. H Broughton, P. A. Bruckman de Renstrom, D. Bruncko, A. Bruni, G. Bruni, L. S. Bruni, S. Bruno, B. H. Brunt, M. Bruschi, N. Bruscino, P. Bryant, L. Bryngemark, T. Buanes, Q. Buat, P. Buchholz, A. G. Buckley, I. A. Budagov, F. Buehrer, M. K. Bugge, O. Bulekov, D. Bullock, T. J. Burch, S. Burdin, C. D. Burgard, A. M. Burger, B. Burghgrave, K. Burka, S. Burke, I. Burmeister, J. T. P. Burr, E. Busato, D. Büscher, V. Büscher, P. Bussey, J. M. Butler, C. M. Buttar, J. M. Butterworth, P. Butti, W. Buttinger, A. Buzatu, A. R. Buzykaev, S. Cabrera Urbán, D. Caforio, H. Cai, V. M. Cairo, O. Cakir, N. Calace, P. Calafiura, A. Calandri, G. Calderini, P. Calfayan, G. Callea, L. P. Caloba, S. Calvente Lopez, D. Calvet, S. Calvet, T. P. Calvet, R. Camacho Toro, S. Camarda, P. Camarri, D. Cameron, R. Caminal Armadans, C. Camincher, S. Campana, M. Campanelli, A. Camplani, A. Campoverde, V. Canale, M. Cano Bret, J. Cantero, T. Cao, M. D. M. Capeans Garrido, I. Caprini, M. Caprini, M. Capua, R. M. Carbone, R. Cardarelli, F. Cardillo, I. Carli, T. Carli, G. Carlino, B. T. Carlson, L. Carminati, R. M. D. Carney, S. Caron, E. Carquin, S. Carrá, G. D. Carrillo-Montoya, D. Casadei, M. P. Casado, A. F. Casha, M. Casolino, D. W. Casper, R. Castelijn, V. Castillo Gimenez, N. F. Castro, A. Catinaccio, J. R. Catmore, A. Cattai, J. Caudron, V. Cavaliere, E. Cavallaro, D. Cavalli, M. Cavalli-Sforza, V. Cavasinni, E. Celebi, F. Ceradini, L. Cerda Alberich, A. S. Cerqueira, A. Cerri, L. Cerrito, F. Cerutti, A. Cervelli, S. A. Cetin, A. Chafaq, D. Chakraborty, S. K. Chan, W. S. Chan, Y. L. Chan, P. Chang, J. D. Chapman, D. G. Charlton, C. C. Chau, C. A. Chavez Barajas, S. Che, S. Cheatham, A. Chegwidden, S. Chekanov, S. V. Chekulaev, G. A. Chelkov, M. A. Chelstowska, C. Chen, C. Chen, H. Chen, J. Chen, S. Chen, S. Chen, X. Chen, Y. Chen, H. C. Cheng, H. J. Cheng, A. Cheplakov, E. Cheremushkina, R. Cherkaoui El Moursli, E. Cheu, K. Cheung, L. Chevalier, V. Chiarella, G. Chiarelli, G. Chiodini, A. S. Chisholm, A. Chitan, Y. H. Chiu, M. V. Chizhov, K. Choi, A. R. Chomont, S. Chouridou, Y. S. Chow, V. Christodoulou, M. C. Chu, J. Chudoba, A. J. Chuinard, J. J. Chwastowski, L. Chytka, A. K. Ciftci, D. Cinca, V. Cindro, I. A. Cioara, A. Ciocio, F. Cirotto, Z. H. Citron, M. Citterio, M. Ciubancan, A. Clark, B. L. Clark, M. R. Clark, P. J. Clark, R. N. Clarke, C. Clement, Y. Coadou, M. Cobal, A. Coccaro, J. Cochran, L. Colasurdo, B. Cole, A. P. Colijn, J. Collot, T. Colombo, P. Conde Muiño, E. Coniavitis, S. H. Connell, I. A. Connelly, S. Constantinescu, G. Conti, F. Conventi, M. Cooke, A. M. Cooper-Sarkar, F. Cormier, K. J. R. Cormier, M. Corradi, F. Corriveau, A. Cortes-Gonzalez, G. Costa, M. J. Costa, D. Costanzo, G. Cottin, G. Cowan, B. E. Cox, K. Cranmer, S. J. Crawley, R. A. Creager, G. Cree, S. Crépé-Renaudin, F. Crescioli, W. A. Cribbs, M. Cristinziani, V. Croft, G. Crosetti, A. Cueto, T. Cuhadar Donszelmann, A. R. Cukierman, J. Cummings, M. Curatolo, J. Cúth, S. Czekierda, P. Czodrowski, G. D’amen, S. D’Auria, L. D’eramo, M. D’Onofrio, M. J. Da Cunha Sargedas De Sousa, C. Da Via, W. Dabrowski, T. Dado, T. Dai, O. Dale, F. Dallaire, C. Dallapiccola, M. Dam, J. R. Dandoy, M. F. Daneri, N. P. Dang, A. C. Daniells, N. S. Dann, M. Danninger, M. Dano Hoffmann, V. Dao, G. Darbo, S. Darmora, J. Dassoulas, A. Dattagupta, T. Daubney, W. Davey, C. David, T. Davidek, D. R. Davis, P. Davison, E. Dawe, I. Dawson, K. De, R. de Asmundis, A. De Benedetti, S. De Castro, S. De Cecco, N. De Groot, P. de Jong, H. De la Torre, F. De Lorenzi, A. De Maria, D. De Pedis, A. De Salvo, U. De Sanctis, A. De Santo, K. De Vasconcelos Corga, J. B. De Vivie De Regie, R. Debbe, C. Debenedetti, D. V. Dedovich, N. Dehghanian, I. Deigaard, M. Del Gaudio, J. Del Peso, D. Delgove, F. Deliot, C. M. Delitzsch, A. Dell’Acqua, L. Dell’Asta, M. Dell’Orso, M. Della Pietra, D. della Volpe, M. Delmastro, C. Delporte, P. A. Delsart, D. A. DeMarco, S. Demers, M. Demichev, A. Demilly, S. P. Denisov, D. Denysiuk, D. Derendarz, J. E. Derkaoui, F. Derue, P. Dervan, K. Desch, C. Deterre, K. Dette, M. R. Devesa, P. O. Deviveiros, A. Dewhurst, S. Dhaliwal, F. A. Di Bello, A. Di Ciaccio, L. Di Ciaccio, W. K. Di Clemente, C. Di Donato, A. Di Girolamo, B. Di Girolamo, B. Di Micco, R. Di Nardo, K. F. Di Petrillo, A. Di Simone, R. Di Sipio, D. Di Valentino, C. Diaconu, M. Diamond, F. A. Dias, M. A. Diaz, E. B. Diehl, J. Dietrich, S. Díez Cornell, A. Dimitrievska, J. Dingfelder, P. Dita, S. Dita, F. Dittus, F. Djama, T. Djobava, J. I. Djuvsland, M. A. B. do Vale, D. Dobos, M. Dobre, D. Dodsworth, C. Doglioni, J. Dolejsi, Z. Dolezal, M. Donadelli, S. Donati, P. Dondero, J. Donini, J. Dopke, A. Doria, M. T. Dova, A. T. Doyle, E. Drechsler, M. Dris, Y. Du, J. Duarte-Campderros, F. Dubinin, A. Dubreuil, E. Duchovni, G. Duckeck, A. Ducourthial, O. A. Ducu, D. Duda, A. Dudarev, A. Chr. Dudder, E. M. Duffield, L. Duflot, M. Dührssen, C. Dulsen, M. Dumancic, A. E. Dumitriu, A. K. Duncan, M. Dunford, A. Duperrin, H. Duran Yildiz, M. Düren, A. Durglishvili, D. Duschinger, B. Dutta, D. Duvnjak, M. Dyndal, B. S. Dziedzic, C. Eckardt, K. M. Ecker, R. C. Edgar, T. Eifert, G. Eigen, K. Einsweiler, T. Ekelof, M. El Kacimi, R. El Kosseifi, V. Ellajosyula, M. Ellert, S. Elles, F. Ellinghaus, A. A. Elliot, N. Ellis, J. Elmsheuser, M. Elsing, D. Emeliyanov, Y. Enari, J. S. Ennis, M. B. Epland, J. Erdmann, A. Ereditato, M. Ernst, S. Errede, M. Escalier, C. Escobar, B. Esposito, O. Estrada Pastor, A. I. Etienvre, E. Etzion, H. Evans, A. Ezhilov, M. Ezzi, F. Fabbri, L. Fabbri, V. Fabiani, G. Facini, R. M. Fakhrutdinov, S. Falciano, R. J. Falla, J. Faltova, Y. Fang, M. Fanti, A. Farbin, A. Farilla, C. Farina, E. M. Farina, T. Farooque, S. Farrell, S. M. Farrington, P. Farthouat, F. Fassi, P. Fassnacht, D. Fassouliotis, M. Faucci Giannelli, A. Favareto, W. J. Fawcett, L. Fayard, O. L. Fedin, W. Fedorko, S. Feigl, L. Feligioni, C. Feng, E. J. Feng, M. J. Fenton, A. B. Fenyuk, L. Feremenga, P. Fernandez Martinez, J. Ferrando, A. Ferrari, P. Ferrari, R. Ferrari, D. E. Ferreira de Lima, A. Ferrer, D. Ferrere, C. Ferretti, F. Fiedler, A. Filipčič, M. Filipuzzi, F. Filthaut, M. Fincke-Keeler, K. D. Finelli, M. C. N. Fiolhais, L. Fiorini, A. Fischer, C. Fischer, J. Fischer, W. C. Fisher, N. Flaschel, I. Fleck, P. Fleischmann, R. R. M. Fletcher, T. Flick, B. M. Flierl, L. R. Flores Castillo, M. J. Flowerdew, G. T. Forcolin, A. Formica, F. A. Förster, A. Forti, A. G. Foster, D. Fournier, H. Fox, S. Fracchia, P. Francavilla, M. Franchini, S. Franchino, D. Francis, L. Franconi, M. Franklin, M. Frate, M. Fraternali, D. Freeborn, S. M. Fressard-Batraneanu, B. Freund, D. Froidevaux, J. A. Frost, C. Fukunaga, T. Fusayasu, J. Fuster, O. Gabizon, A. Gabrielli, A. Gabrielli, G. P. Gach, S. Gadatsch, S. Gadomski, G. Gagliardi, L. G. Gagnon, C. Galea, B. Galhardo, E. J. Gallas, B. J. Gallop, P. Gallus, G. Galster, K. K. Gan, S. Ganguly, Y. Gao, Y. S. Gao, F. M. Garay Walls, C. García, J. E. García Navarro, J. A. García Pascual, M. Garcia-Sciveres, R. W. Gardner, N. Garelli, V. Garonne, A. Gascon Bravo, K. Gasnikova, C. Gatti, A. Gaudiello, G. Gaudio, I. L. Gavrilenko, C. Gay, G. Gaycken, E. N. Gazis, C. N. P. Gee, J. Geisen, M. Geisen, M. P. Geisler, K. Gellerstedt, C. Gemme, M. H. Genest, C. Geng, S. Gentile, C. Gentsos, S. George, D. Gerbaudo, G. Geßner, S. Ghasemi, M. Ghneimat, B. Giacobbe, S. Giagu, N. Giangiacomi, P. Giannetti, S. M. Gibson, M. Gignac, M. Gilchriese, D. Gillberg, G. Gilles, D. M. Gingrich, M. P. Giordani, F. M. Giorgi, P. F. Giraud, P. Giromini, G. Giugliarelli, D. Giugni, F. Giuli, C. Giuliani, M. Giulini, B. K. Gjelsten, S. Gkaitatzis, I. Gkialas, E. L. Gkougkousis, P. Gkountoumis, L. K. Gladilin, C. Glasman, J. Glatzer, P. C. F. Glaysher, A. Glazov, M. Goblirsch-Kolb, J. Godlewski, S. Goldfarb, T. Golling, D. Golubkov, A. Gomes, R. Gonçalo, R. Goncalves Gama, J. Goncalves Pinto Firmino Da Costa, G. Gonella, L. Gonella, A. Gongadze, J. L. Gonski, S. González de la Hoz, S. Gonzalez-Sevilla, L. Goossens, P. A. Gorbounov, H. A. Gordon, I. Gorelov, B. Gorini, E. Gorini, A. Gorišek, A. T. Goshaw, C. Gössling, M. I. Gostkin, C. A. Gottardo, C. R. Goudet, D. Goujdami, A. G. Goussiou, N. Govender, E. Gozani, I. Grabowska-Bold, P. O. J. Gradin, J. Gramling, E. Gramstad, S. Grancagnolo, V. Gratchev, P. M. Gravila, C. Gray, H. M. Gray, Z. D. Greenwood, C. Grefe, K. Gregersen, I. M. Gregor, P. Grenier, K. Grevtsov, J. Griffiths, A. A. Grillo, K. Grimm, S. Grinstein, Ph. Gris, J.-F. Grivaz, S. Groh, E. Gross, J. Grosse-Knetter, G. C. Grossi, Z. J. Grout, A. Grummer, L. Guan, W. Guan, J. Guenther, F. Guescini, D. Guest, O. Gueta, B. Gui, E. Guido, T. Guillemin, S. Guindon, U. Gul, C. Gumpert, J. Guo, W. Guo, Y. Guo, R. Gupta, S. Gurbuz, G. Gustavino, B. J. Gutelman, P. Gutierrez, N. G. Gutierrez Ortiz, C. Gutschow, C. Guyot, M. P. Guzik, C. Gwenlan, C. B. Gwilliam, A. Haas, C. Haber, H. K. Hadavand, N. Haddad, A. Hadef, S. Hageböck, M. Hagihara, H. Hakobyan, M. Haleem, J. Haley, G. Halladjian, G. D. Hallewell, K. Hamacher, P. Hamal, K. Hamano, A. Hamilton, G. N. Hamity, P. G. Hamnett, L. Han, S. Han, K. Hanagaki, K. Hanawa, M. Hance, D. M. Handl, B. Haney, P. Hanke, J. B. Hansen, J. D. Hansen, M. C. Hansen, P. H. Hansen, K. Hara, A. S. Hard, T. Harenberg, F. Hariri, S. Harkusha, P. F. Harrison, N. M. Hartmann, Y. Hasegawa, A. Hasib, S. Hassani, S. Haug, R. Hauser, L. Hauswald, L. B. Havener, M. Havranek, C. M. Hawkes, R. J. Hawkings, D. Hayakawa, D. Hayden, C. P. Hays, J. M. Hays, H. S. Hayward, S. J. Haywood, S. J. Head, T. Heck, V. Hedberg, L. Heelan, S. Heer, K. K. Heidegger, S. Heim, T. Heim, B. Heinemann, J. J. Heinrich, L. Heinrich, C. Heinz, J. Hejbal, L. Helary, A. Held, S. Hellman, C. Helsens, R. C. W. Henderson, Y. Heng, S. Henkelmann, A. M. Henriques Correia, S. Henrot-Versille, G. H. Herbert, H. Herde, V. Herget, Y. Hernández Jiménez, H. Herr, G. Herten, R. Hertenberger, L. Hervas, T. C. Herwig, G. G. Hesketh, N. P. Hessey, J. W. Hetherly, S. Higashino, E. Higón-Rodriguez, K. Hildebrand, E. Hill, J. C. Hill, K. H. Hiller, S. J. Hillier, M. Hils, I. Hinchliffe, M. Hirose, D. Hirschbuehl, B. Hiti, O. Hladik, D. R. Hlaluku, X. Hoad, J. Hobbs, N. Hod, M. C. Hodgkinson, P. Hodgson, A. Hoecker, M. R. Hoeferkamp, F. Hoenig, D. Hohn, T. R. Holmes, M. Homann, S. Honda, T. Honda, T. M. Hong, B. H. Hooberman, W. H. Hopkins, Y. Horii, A. J. Horton, J.-Y. Hostachy, A. Hostiuc, S. Hou, A. Hoummada, J. Howarth, J. Hoya, M. Hrabovsky, J. Hrdinka, I. Hristova, J. Hrivnac, T. Hryn’ova, A. Hrynevich, P. J. Hsu, S.-C. Hsu, Q. Hu, S. Hu, Y. Huang, Z. Hubacek, F. Hubaut, F. Huegging, T. B. Huffman, E. W. Hughes, M. Huhtinen, R. F. H. Hunter, P. Huo, N. Huseynov, J. Huston, J. Huth, R. Hyneman, G. Iacobucci, G. Iakovidis, I. Ibragimov, L. Iconomidou-Fayard, Z. Idrissi, P. Iengo, O. Igonkina, T. Iizawa, Y. Ikegami, M. Ikeno, Y. Ilchenko, D. Iliadis, N. Ilic, F. Iltzsche, G. Introzzi, P. Ioannou, M. Iodice, K. Iordanidou, V. Ippolito, M. F. Isacson, N. Ishijima, M. Ishino, M. Ishitsuka, C. Issever, S. Istin, F. Ito, J. M. Iturbe Ponce, R. Iuppa, H. Iwasaki, J. M. Izen, V. Izzo, S. Jabbar, P. Jackson, R. M. Jacobs, V. Jain, K. B. Jakobi, K. Jakobs, S. Jakobsen, T. Jakoubek, D. O. Jamin, D. K. Jana, R. Jansky, J. Janssen, M. Janus, P. A. Janus, G. Jarlskog, N. Javadov, T. Javůrek, M. Javurkova, F. Jeanneau, L. Jeanty, J. Jejelava, A. Jelinskas, P. Jenni, C. Jeske, S. Jézéquel, H. Ji, J. Jia, H. Jiang, Y. Jiang, Z. Jiang, S. Jiggins, J. Jimenez Pena, S. Jin, A. Jinaru, O. Jinnouchi, H. Jivan, P. Johansson, K. A. Johns, C. A. Johnson, W. J. Johnson, K. Jon-And, R. W. L. Jones, S. D. Jones, S. Jones, T. J. Jones, J. Jongmanns, P. M. Jorge, J. Jovicevic, X. Ju, A. Juste Rozas, M. K. Köhler, A. Kaczmarska, M. Kado, H. Kagan, M. Kagan, S. J. Kahn, T. Kaji, E. Kajomovitz, C. W. Kalderon, A. Kaluza, S. Kama, A. Kamenshchikov, N. Kanaya, L. Kanjir, V. A. Kantserov, J. Kanzaki, B. Kaplan, L. S. Kaplan, D. Kar, K. Karakostas, N. Karastathis, M. J. Kareem, E. Karentzos, S. N. Karpov, Z. M. Karpova, K. Karthik, V. Kartvelishvili, A. N. Karyukhin, K. Kasahara, L. Kashif, R. D. Kass, A. Kastanas, Y. Kataoka, C. Kato, A. Katre, J. Katzy, K. Kawade, K. Kawagoe, T. Kawamoto, G. Kawamura, E. F. Kay, V. F. Kazanin, R. Keeler, R. Kehoe, J. S. Keller, E. Kellermann, J. J. Kempster, J Kendrick, H. Keoshkerian, O. Kepka, B. P. Kerševan, S. Kersten, R. A. Keyes, M. Khader, F. Khalil-zada, A. Khanov, A. G. Kharlamov, T. Kharlamova, A. Khodinov, T. J. Khoo, V. Khovanskiy, E. Khramov, J. Khubua, S. Kido, C. R. Kilby, H. Y. Kim, S. H. Kim, Y. K. Kim, N. Kimura, O. M. Kind, B. T. King, D. Kirchmeier, J. Kirk, A. E. Kiryunin, T. Kishimoto, D. Kisielewska, V. Kitali, O. Kivernyk, E. Kladiva, T. Klapdor-Kleingrothaus, M. H. Klein, M. Klein, U. Klein, K. Kleinknecht, P. Klimek, A. Klimentov, R. Klingenberg, T. Klingl, T. Klioutchnikova, E.-E. Kluge, P. Kluit, S. Kluth, E. Kneringer, E. B. F. G. Knoops, A. Knue, A. Kobayashi, D. Kobayashi, T. Kobayashi, M. Kobel, M. Kocian, P. Kodys, T. Koffas, E. Koffeman, N. M. Köhler, T. Koi, M. Kolb, I. Koletsou, A. A. Komar, T. Kondo, N. Kondrashova, K. Köneke, A. C. König, T. Kono, R. Konoplich, N. Konstantinidis, R. Kopeliansky, S. Koperny, A. K. Kopp, K. Korcyl, K. Kordas, A. Korn, A. A. Korol, I. Korolkov, E. V. Korolkova, O. Kortner, S. Kortner, T. Kosek, V. V. Kostyukhin, A. Kotwal, A. Koulouris, A. Kourkoumeli-Charalampidi, C. Kourkoumelis, E. Kourlitis, V. Kouskoura, A. B. Kowalewska, R. Kowalewski, T. Z. Kowalski, C. Kozakai, W. Kozanecki, A. S. Kozhin, V. A. Kramarenko, G. Kramberger, D. Krasnopevtsev, M. W. Krasny, A. Krasznahorkay, D. Krauss, J. A. Kremer, J. Kretzschmar, K. Kreutzfeldt, P. Krieger, K. Krizka, K. Kroeninger, H. Kroha, J. Kroll, J. Kroll, J. Kroseberg, J. Krstic, U. Kruchonak, H. Krüger, N. Krumnack, M. C. Kruse, T. Kubota, H. Kucuk, S. Kuday, J. T. Kuechler, S. Kuehn, A. Kugel, F. Kuger, T. Kuhl, V. Kukhtin, R. Kukla, Y. Kulchitsky, S. Kuleshov, Y. P. Kulinich, M. Kuna, T. Kunigo, A. Kupco, T. Kupfer, O. Kuprash, H. Kurashige, L. L. Kurchaninov, Y. A. Kurochkin, M. G. Kurth, E. S. Kuwertz, M. Kuze, J. Kvita, T. Kwan, D. Kyriazopoulos, A. La Rosa, J. L. La Rosa Navarro, L. La Rotonda, F. La Ruffa, C. Lacasta, F. Lacava, J. Lacey, D. P. J. Lack, H. Lacker, D. Lacour, E. Ladygin, R. Lafaye, B. Laforge, T. Lagouri, S. Lai, S. Lammers, W. Lampl, E. Lançon, U. Landgraf, M. P. J. Landon, M. C. Lanfermann, V. S. Lang, J. C. Lange, R. J. Langenberg, A. J. Lankford, F. Lanni, K. Lantzsch, A. Lanza, A. Lapertosa, S. Laplace, J. F. Laporte, T. Lari, F. Lasagni Manghi, M. Lassnig, T. S. Lau, P. Laurelli, W. Lavrijsen, A. T. Law, P. Laycock, T. Lazovich, M. Lazzaroni, B. Le, O. Le Dortz, E. Le Guirriec, E. P. Le Quilleuc, M. LeBlanc, T. LeCompte, F. Ledroit-Guillon, C. A. Lee, G. R. Lee, S. C. Lee, L. Lee, B. Lefebvre, G. Lefebvre, M. Lefebvre, F. Legger, C. Leggett, G. Lehmann Miotto, X. Lei, W. A. Leight, M. A. L. Leite, R. Leitner, D. Lellouch, B. Lemmer, K. J. C. Leney, T. Lenz, B. Lenzi, R. Leone, S. Leone, C. Leonidopoulos, G. Lerner, C. Leroy, R. Les, A. A. J. Lesage, C. G. Lester, M. Levchenko, J. Levêque, D. Levin, L. J. Levinson, M. Levy, D. Lewis, B. Li, Changqiao Li, H. Li, L. Li, Q. Li, Q. Li, S. Li, X. Li, Y. Li, Z. Liang, B. Liberti, A. Liblong, K. Lie, J. Liebal, W. Liebig, A. Limosani, K. Lin, S. C. Lin, T. H. Lin, R. A. Linck, B. E. Lindquist, A. E. Lionti, E. Lipeles, A. Lipniacka, M. Lisovyi, T. M. Liss, A. Lister, A. M. Litke, B. Liu, H. Liu, H. Liu, J. K. K. Liu, J. Liu, J. B. Liu, K. Liu, L. Liu, M. Liu, Y. L. Liu, Y. Liu, M. Livan, A. Lleres, J. Llorente Merino, S. L. Lloyd, C. Y. Lo, F. Lo Sterzo, E. M. Lobodzinska, P. Loch, F. K. Loebinger, A. Loesle, K. M. Loew, T. Lohse, K. Lohwasser, M. Lokajicek, B. A. Long, J. D. Long, R. E. Long, L. Longo, K. A. Looper, J. A. Lopez, I. Lopez Paz, A. Lopez Solis, J. Lorenz, N. Lorenzo Martinez, M. Losada, P. J. Lösel, X. Lou, A. Lounis, J. Love, P. A. Love, H. Lu, N. Lu, Y. J. Lu, H. J. Lubatti, C. Luci, A. Lucotte, C. Luedtke, F. Luehring, W. Lukas, L. Luminari, O. Lundberg, B. Lund-Jensen, M. S. Lutz, P. M. Luzi, D. Lynn, R. Lysak, E. Lytken, F. Lyu, V. Lyubushkin, H. Ma, L. L. Ma, Y. Ma, G. Maccarrone, A. Macchiolo, C. M. Macdonald, B. Maček, J. Machado Miguens, D. Madaffari, R. Madar, W. F. Mader, A. Madsen, N. Madysa, J. Maeda, S. Maeland, T. Maeno, A. S. Maevskiy, V. Magerl, C. Maiani, C. Maidantchik, T. Maier, A. Maio, O. Majersky, S. Majewski, Y. Makida, N. Makovec, B. Malaescu, Pa. Malecki, V. P. Maleev, F. Malek, U. Mallik, D. Malon, C. Malone, S. Maltezos, S. Malyukov, J. Mamuzic, G. Mancini, I. Mandić, J. Maneira, L. Manhaes de Andrade Filho, J. Manjarres Ramos, K. H. Mankinen, A. Mann, A. Manousos, B. Mansoulie, J. D. Mansour, R. Mantifel, M. Mantoani, S. Manzoni, L. Mapelli, G. Marceca, L. March, L. Marchese, G. Marchiori, M. Marcisovsky, C. A. Marin Tobon, M. Marjanovic, D. E. Marley, F. Marroquim, S. P. Marsden, Z. Marshall, M. U. F Martensson, S. Marti-Garcia, C. B. Martin, T. A. Martin, V. J. Martin, B. Martin dit Latour, M. Martinez, V. I. Martinez Outschoorn, S. Martin-Haugh, V. S. Martoiu, A. C. Martyniuk, A. Marzin, L. Masetti, T. Mashimo, R. Mashinistov, J. Masik, A. L. Maslennikov, L. H. Mason, L. Massa, P. Mastrandrea, A. Mastroberardino, T. Masubuchi, P. Mättig, J. Maurer, S. J. Maxfield, D. A. Maximov, R. Mazini, I. Maznas, S. M. Mazza, N. C. Mc Fadden, G. Mc Goldrick, S. P. Mc Kee, A. McCarn, R. L. McCarthy, T. G. McCarthy, L. I. McClymont, E. F. McDonald, J. A. Mcfayden, G. Mchedlidze, S. J. McMahon, P. C. McNamara, C. J. McNicol, R. A. McPherson, S. Meehan, T. J. Megy, S. Mehlhase, A. Mehta, T. Meideck, K. Meier, B. Meirose, D. Melini, B. R. Mellado Garcia, J. D. Mellenthin, M. Melo, F. Meloni, A. Melzer, S. B. Menary, L. Meng, X. T. Meng, A. Mengarelli, S. Menke, E. Meoni, S. Mergelmeyer, C. Merlassino, P. Mermod, L. Merola, C. Meroni, F. S. Merritt, A. Messina, J. Metcalfe, A. S. Mete, C. Meyer, J.-P. Meyer, J. Meyer, H. Meyer Zu Theenhausen, F. Miano, R. P. Middleton, S. Miglioranzi, L. Mijović, G. Mikenberg, M. Mikestikova, M. Mikuž, M. Milesi, A. Milic, D. A. Millar, D. W. Miller, C. Mills, A. Milov, D. A. Milstead, A. A. Minaenko, Y. Minami, I. A. Minashvili, A. I. Mincer, B. Mindur, M. Mineev, Y. Minegishi, Y. Ming, L. M. Mir, A. Mirto, K. P. Mistry, T. Mitani, J. Mitrevski, V. A. Mitsou, A. Miucci, P. S. Miyagawa, A. Mizukami, J. U. Mjörnmark, T. Mkrtchyan, M. Mlynarikova, T. Moa, K. Mochizuki, P. Mogg, S. Mohapatra, S. Molander, R. Moles-Valls, M. C. Mondragon, K. Mönig, J. Monk, E. Monnier, A. Montalbano, J. Montejo Berlingen, F. Monticelli, S. Monzani, R. W. Moore, N. Morange, D. Moreno, M. Moreno Llácer, P. Morettini, S. Morgenstern, D. Mori, T. Mori, M. Morii, M. Morinaga, V. Morisbak, A. K. Morley, G. Mornacchi, J. D. Morris, L. Morvaj, P. Moschovakos, M. Mosidze, H. J. Moss, J. Moss, K. Motohashi, R. Mount, E. Mountricha, E. J. W. Moyse, S. Muanza, F. Mueller, J. Mueller, R. S. P. Mueller, D. Muenstermann, P. Mullen, G. A. Mullier, F. J. Munoz Sanchez, W. J. Murray, H. Musheghyan, M. Muškinja, A. G. Myagkov, M. Myska, B. P. Nachman, O. Nackenhorst, K. Nagai, R. Nagai, K. Nagano, Y. Nagasaka, K. Nagata, M. Nagel, E. Nagy, A. M. Nairz, Y. Nakahama, K. Nakamura, T. Nakamura, I. Nakano, R. F. Naranjo Garcia, R. Narayan, D. I. Narrias Villar, I. Naryshkin, T. Naumann, G. Navarro, R. Nayyar, H. A. Neal, P. Yu. Nechaeva, T. J. Neep, A. Negri, M. Negrini, S. Nektarijevic, C. Nellist, A. Nelson, M. E. Nelson, S. Nemecek, P. Nemethy, M. Nessi, M. S. Neubauer, M. Neumann, P. R. Newman, T. Y. Ng, T. Nguyen Manh, R. B. Nickerson, R. Nicolaidou, J. Nielsen, N. Nikiforou, V. Nikolaenko, I. Nikolic-Audit, K. Nikolopoulos, J. K. Nilsen, P. Nilsson, Y. Ninomiya, A. Nisati, N. Nishu, R. Nisius, I. Nitsche, T. Nitta, T. Nobe, Y. Noguchi, M. Nomachi, I. Nomidis, M. A. Nomura, T. Nooney, M. Nordberg, N. Norjoharuddeen, O. Novgorodova, M. Nozaki, L. Nozka, K. Ntekas, E. Nurse, F. Nuti, K. O’connor, D. C. O’Neil, A. A. O’Rourke, V. O’Shea, F. G. Oakham, H. Oberlack, T. Obermann, J. Ocariz, A. Ochi, I. Ochoa, J. P. Ochoa-Ricoux, S. Oda, S. Odaka, A. Oh, S. H. Oh, C. C. Ohm, H. Ohman, H. Oide, H. Okawa, Y. Okumura, T. Okuyama, A. Olariu, L. F. Oleiro Seabra, S. A. Olivares Pino, D. Oliveira Damazio, A. Olszewski, J. Olszowska, A. Onofre, K. Onogi, P. U. E. Onyisi, H. Oppen, M. J. Oreglia, Y. Oren, D. Orestano, N. Orlando, R. S. Orr, B. Osculati, R. Ospanov, G. Otero y Garzon, H. Otono, M. Ouchrif, F. Ould-Saada, A. Ouraou, K. P. Oussoren, Q. Ouyang, M. Owen, R. E. Owen, V. E. Ozcan, N. Ozturk, K. Pachal, A. Pacheco Pages, L. Pacheco Rodriguez, C. Padilla Aranda, S. Pagan Griso, M. Paganini, F. Paige, G. Palacino, S. Palazzo, S. Palestini, M. Palka, D. Pallin, E. St. Panagiotopoulou, I. Panagoulias, C. E. Pandini, J. G. Panduro Vazquez, P. Pani, S. Panitkin, D. Pantea, L. Paolozzi, Th. D. Papadopoulou, K. Papageorgiou, A. Paramonov, D. Paredes Hernandez, A. J. Parker, M. A. Parker, K. A. Parker, F. Parodi, J. A. Parsons, U. Parzefall, V. R. Pascuzzi, J. M. Pasner, E. Pasqualucci, S. Passaggio, Fr. Pastore, S. Pataraia, J. R. Pater, T. Pauly, B. Pearson, S. Pedraza Lopez, R. Pedro, S. V. Peleganchuk, O. Penc, C. Peng, H. Peng, J. Penwell, B. S. Peralva, M. M. Perego, D. V. Perepelitsa, F. Peri, L. Perini, H. Pernegger, S. Perrella, R. Peschke, V. D. Peshekhonov, K. Peters, R. F. Y. Peters, B. A. Petersen, T. C. Petersen, E. Petit, A. Petridis, C. Petridou, P. Petroff, E. Petrolo, M. Petrov, F. Petrucci, N. E. Pettersson, A. Peyaud, R. Pezoa, F. H. Phillips, P. W. Phillips, G. Piacquadio, E. Pianori, A. Picazio, M. A. Pickering, R. Piegaia, J. E. Pilcher, A. D. Pilkington, M. Pinamonti, J. L. Pinfold, H. Pirumov, M. Pitt, L. Plazak, M.-A. Pleier, V. Pleskot, E. Plotnikova, D. Pluth, P. Podberezko, R. Poettgen, R. Poggi, L. Poggioli, I. Pogrebnyak, D. Pohl, I. Pokharel, G. Polesello, A. Poley, A. Policicchio, R. Polifka, A. Polini, C. S. Pollard, V. Polychronakos, K. Pommès, D. Ponomarenko, L. Pontecorvo, G. A. Popeneciu, D. M. Portillo Quintero, S. Pospisil, K. Potamianos, I. N. Potrap, C. J. Potter, H. Potti, T. Poulsen, J. Poveda, M. E. Pozo Astigarraga, P. Pralavorio, A. Pranko, S. Prell, D. Price, M. Primavera, S. Prince, N. Proklova, K. Prokofiev, F. Prokoshin, S. Protopopescu, J. Proudfoot, M. Przybycien, A. Puri, P. Puzo, J. Qian, G. Qin, Y. Qin, A. Quadt, M. Queitsch-Maitland, D. Quilty, S. Raddum, V. Radeka, V. Radescu, S. K. Radhakrishnan, P. Radloff, P. Rados, F. Ragusa, G. Rahal, J. A. Raine, S. Rajagopalan, C. Rangel-Smith, T. Rashid, S. Raspopov, M. G. Ratti, D. M. Rauch, F. Rauscher, S. Rave, I. Ravinovich, J. H. Rawling, M. Raymond, A. L. Read, N. P. Readioff, M. Reale, D. M. Rebuzzi, A. Redelbach, G. Redlinger, R. Reece, R. G. Reed, K. Reeves, L. Rehnisch, J. Reichert, A. Reiss, C. Rembser, H. Ren, M. Rescigno, S. Resconi, E. D. Resseguie, S. Rettie, E. Reynolds, O. L. Rezanova, P. Reznicek, R. Rezvani, R. Richter, S. Richter, E. Richter-Was, O. Ricken, M. Ridel, P. Rieck, C. J. Riegel, J. Rieger, O. Rifki, M. Rijssenbeek, A. Rimoldi, M. Rimoldi, L. Rinaldi, G. Ripellino, B. Ristić, E. Ritsch, I. Riu, F. Rizatdinova, E. Rizvi, C. Rizzi, R. T. Roberts, S. H. Robertson, A. Robichaud-Veronneau, D. Robinson, J. E. M. Robinson, A. Robson, E. Rocco, C. Roda, Y. Rodina, S. Rodriguez Bosca, A. Rodriguez Perez, D. Rodriguez Rodriguez, S. Roe, C. S. Rogan, O. Røhne, J. Roloff, A. Romaniouk, M. Romano, S. M. Romano Saez, E. Romero Adam, N. Rompotis, M. Ronzani, L. Roos, S. Rosati, K. Rosbach, P. Rose, N.-A. Rosien, E. Rossi, L. P. Rossi, J. H. N. Rosten, R. Rosten, M. Rotaru, J. Rothberg, D. Rousseau, A. Rozanov, Y. Rozen, X. Ruan, F. Rubbo, E. M. Ruettinger, F. Rühr, A. Ruiz-Martinez, Z. Rurikova, N. A. Rusakovich, H. L. Russell, J. P. Rutherfoord, N. Ruthmann, Y. F. Ryabov, M. Rybar, G. Rybkin, S. Ryu, A. Ryzhov, G. F. Rzehorz, A. F. Saavedra, G. Sabato, S. Sacerdoti, H. F.-W. Sadrozinski, R. Sadykov, F. Safai Tehrani, P. Saha, M. Sahinsoy, M. Saimpert, M. Saito, T. Saito, H. Sakamoto, Y. Sakurai, G. Salamanna, J. E. Salazar Loyola, D. Salek, P. H. Sales De Bruin, D. Salihagic, A. Salnikov, J. Salt, D. Salvatore, F. Salvatore, A. Salvucci, A. Salzburger, D. Sammel, D. Sampsonidis, D. Sampsonidou, J. Sánchez, V. Sanchez Martinez, A. Sanchez Pineda, H. Sandaker, R. L. Sandbach, C. O. Sander, M. Sandhoff, C. Sandoval, D. P. C. Sankey, M. Sannino, Y. Sano, A. Sansoni, C. Santoni, H. Santos, I. Santoyo Castillo, A. Sapronov, J. G. Saraiva, B. Sarrazin, O. Sasaki, K. Sato, E. Sauvan, G. Savage, P. Savard, N. Savic, C. Sawyer, L. Sawyer, J. Saxon, C. Sbarra, A. Sbrizzi, T. Scanlon, D. A. Scannicchio, J. Schaarschmidt, P. Schacht, B. M. Schachtner, D. Schaefer, L. Schaefer, R. Schaefer, J. Schaeffer, S. Schaepe, S. Schaetzel, U. Schäfer, A. C. Schaffer, D. Schaile, R. D. Schamberger, V. A. Schegelsky, D. Scheirich, M. Schernau, C. Schiavi, S. Schier, L. K. Schildgen, C. Schillo, M. Schioppa, S. Schlenker, K. R. Schmidt-Sommerfeld, K. Schmieden, C. Schmitt, S. Schmitt, S. Schmitz, U. Schnoor, L. Schoeffel, A. Schoening, B. D. Schoenrock, E. Schopf, M. Schott, J. F. P. Schouwenberg, J. Schovancova, S. Schramm, N. Schuh, A. Schulte, M. J. Schultens, H.-C. Schultz-Coulon, H. Schulz, M. Schumacher, B. A. Schumm, Ph. Schune, A. Schwartzman, T. A. Schwarz, H. Schweiger, Ph. Schwemling, R. Schwienhorst, J. Schwindling, A. Sciandra, G. Sciolla, M. Scornajenghi, F. Scuri, F. Scutti, J. Searcy, P. Seema, S. C. Seidel, A. Seiden, J. M. Seixas, G. Sekhniaidze, K. Sekhon, S. J. Sekula, N. Semprini-Cesari, S. Senkin, C. Serfon, L. Serin, L. Serkin, M. Sessa, R. Seuster, H. Severini, T. Sfiligoj, F. Sforza, A. Sfyrla, E. Shabalina, N. W. Shaikh, L. Y. Shan, R. Shang, J. T. Shank, M. Shapiro, P. B. Shatalov, K. Shaw, S. M. Shaw, A. Shcherbakova, C. Y. Shehu, Y. Shen, N. Sherafati, P. Sherwood, L. Shi, S. Shimizu, C. O. Shimmin, M. Shimojima, I. P. J. Shipsey, S. Shirabe, M. Shiyakova, J. Shlomi, A. Shmeleva, D. Shoaleh Saadi, M. J. Shochet, S. Shojaii, D. R. Shope, S. Shrestha, E. Shulga, M. A. Shupe, P. Sicho, A. M. Sickles, P. E. Sidebo, E. Sideras Haddad, O. Sidiropoulou, A. Sidoti, F. Siegert, Dj. Sijacki, J. Silva, S. B. Silverstein, V. Simak, L. Simic, S. Simion, E. Simioni, B. Simmons, M. Simon, P. Sinervo, N. B. Sinev, M. Sioli, G. Siragusa, I. Siral, S. Yu. Sivoklokov, J. Sjölin, M. B. Skinner, P. Skubic, M. Slater, T. Slavicek, M. Slawinska, K. Sliwa, R. Slovak, V. Smakhtin, B. H. Smart, J. Smiesko, N. Smirnov, S. Yu. Smirnov, Y. Smirnov, L. N. Smirnova, O. Smirnova, J. W. Smith, M. N. K. Smith, R. W. Smith, M. Smizanska, K. Smolek, A. A. Snesarev, I. M. Snyder, S. Snyder, R. Sobie, F. Socher, A. Soffer, A. Søgaard, D. A. Soh, G. Sokhrannyi, C. A. Solans Sanchez, M. Solar, E. Yu. Soldatov, U. Soldevila, A. A. Solodkov, A. Soloshenko, O. V. Solovyanov, V. Solovyev, P. Sommer, H. Son, A. Sopczak, D. Sosa, C. L. Sotiropoulou, S. Sottocornola, R. Soualah, A. M. Soukharev, D. South, B. C. Sowden, S. Spagnolo, M. Spalla, M. Spangenberg, F. Spanò, D. Sperlich, F. Spettel, T. M. Spieker, R. Spighi, G. Spigo, L. A. Spiller, M. Spousta, R. D. St. Denis, A. Stabile, R. Stamen, S. Stamm, E. Stanecka, R. W. Stanek, C. Stanescu, M. M. Stanitzki, B. S. Stapf, S. Stapnes, E. A. Starchenko, G. H. Stark, J. Stark, S. H Stark, P. Staroba, P. Starovoitov, S. Stärz, R. Staszewski, M. Stegler, P. Steinberg, B. Stelzer, H. J. Stelzer, O. Stelzer-Chilton, H. Stenzel, T. J. Stevenson, G. A. Stewart, M. C. Stockton, M. Stoebe, G. Stoicea, P. Stolte, S. Stonjek, A. R. Stradling, A. Straessner, M. E. Stramaglia, J. Strandberg, S. Strandberg, M. Strauss, P. Strizenec, R. Ströhmer, D. M. Strom, R. Stroynowski, A. Strubig, S. A. Stucci, B. Stugu, N. A. Styles, D. Su, J. Su, S. Suchek, Y. Sugaya, M. Suk, V. V. Sulin, D. M. S. Sultan, S. Sultansoy, T. Sumida, S. Sun, X. Sun, K. Suruliz, C. J. E. Suster, M. R. Sutton, S. Suzuki, M. Svatos, M. Swiatlowski, S. P. Swift, I. Sykora, T. Sykora, D. Ta, K. Tackmann, J. Taenzer, A. Taffard, R. Tafirout, E. Tahirovic, N. Taiblum, H. Takai, R. Takashima, E. H. Takasugi, K. Takeda, T. Takeshita, Y. Takubo, M. Talby, A. A. Talyshev, J. Tanaka, M. Tanaka, R. Tanaka, S. Tanaka, R. Tanioka, B. B. Tannenwald, S. Tapia Araya, S. Tapprogge, S. Tarem, G. F. Tartarelli, P. Tas, M. Tasevsky, T. Tashiro, E. Tassi, A. Tavares Delgado, Y. Tayalati, A. C. Taylor, A. J. Taylor, G. N. Taylor, P. T. E. Taylor, W. Taylor, P. Teixeira-Dias, D. Temple, H. Ten Kate, P. K. Teng, J. J. Teoh, F. Tepel, S. Terada, K. Terashi, J. Terron, S. Terzo, M. Testa, R. J. Teuscher, S. J. Thais, T. Theveneaux-Pelzer, F. Thiele, J. P. Thomas, J. Thomas-Wilsker, P. D. Thompson, A. S. Thompson, L. A. Thomsen, E. Thomson, Y. Tian, M. J. Tibbetts, R. E. Ticse Torres, V. O. Tikhomirov, Yu. A. Tikhonov, S. Timoshenko, P. Tipton, S. Tisserant, K. Todome, S. Todorova-Nova, S. Todt, J. Tojo, S. Tokár, K. Tokushuku, E. Tolley, L. Tomlinson, M. Tomoto, L. Tompkins, K. Toms, B. Tong, P. Tornambe, E. Torrence, H. Torres, E. Torró Pastor, J. Toth, F. Touchard, D. R. Tovey, C. J. Treado, T. Trefzger, F. Tresoldi, A. Tricoli, I. M. Trigger, S. Trincaz-Duvoid, M. F. Tripiana, W. Trischuk, B. Trocmé, A. Trofymov, C. Troncon, M. Trottier-McDonald, M. Trovatelli, L. Truong, M. Trzebinski, A. Trzupek, K. W. Tsang, J. C.-L. Tseng, P. V. Tsiareshka, G. Tsipolitis, N. Tsirintanis, S. Tsiskaridze, V. Tsiskaridze, E. G. Tskhadadze, I. I. Tsukerman, V. Tsulaia, S. Tsuno, D. Tsybychev, Y. Tu, A. Tudorache, V. Tudorache, T. T. Tulbure, A. N. Tuna, S. Turchikhin, D. Turgeman, I. Turk Cakir, R. Turra, P. M. Tuts, G. Ucchielli, I. Ueda, M. Ughetto, F. Ukegawa, G. Unal, A. Undrus, G. Unel, F. C. Ungaro, Y. Unno, K. Uno, C. Unverdorben, J. Urban, P. Urquijo, P. Urrejola, G. Usai, J. Usui, L. Vacavant, V. Vacek, B. Vachon, K. O. H. Vadla, A. Vaidya, C. Valderanis, E. Valdes Santurio, M. Valente, S. Valentinetti, A. Valero, L. Valéry, S. Valkar, A. Vallier, J. A. Valls Ferrer, W. Van Den Wollenberg, H. van der Graaf, P. van Gemmeren, J. Van Nieuwkoop, I. van Vulpen, M. C. van Woerden, M. Vanadia, W. Vandelli, A. Vaniachine, P. Vankov, G. Vardanyan, R. Vari, E. W. Varnes, C. Varni, T. Varol, D. Varouchas, A. Vartapetian, K. E. Varvell, J. G. Vasquez, G. A. Vasquez, F. Vazeille, D. Vazquez Furelos, T. Vazquez Schroeder, J. Veatch, V. Veeraraghavan, L. M. Veloce, F. Veloso, S. Veneziano, A. Ventura, M. Venturi, N. Venturi, A. Venturini, V. Vercesi, M. Verducci, W. Verkerke, A. T. Vermeulen, J. C. Vermeulen, M. C. Vetterli, N. Viaux Maira, O. Viazlo, I. Vichou, T. Vickey, O. E. Vickey Boeriu, G. H. A. Viehhauser, S. Viel, L. Vigani, M. Villa, M. Villaplana Perez, E. Vilucchi, M. G. Vincter, V. B. Vinogradov, A. Vishwakarma, C. Vittori, I. Vivarelli, S. Vlachos, M. Vogel, P. Vokac, G. Volpi, H. von der Schmitt, E. von Toerne, V. Vorobel, K. Vorobev, M. Vos, R. Voss, J. H. Vossebeld, N. Vranjes, M. Vranjes Milosavljevic, V. Vrba, M. Vreeswijk, R. Vuillermet, I. Vukotic, P. Wagner, W. Wagner, J. Wagner-Kuhr, H. Wahlberg, S. Wahrmund, J. Walder, R. Walker, W. Walkowiak, V. Wallangen, C. Wang, C. Wang, F. Wang, H. Wang, H. Wang, J. Wang, J. Wang, Q. Wang, R.-J. Wang, R. Wang, S. M. Wang, T. Wang, W. Wang, W. Wang, Z. Wang, C. Wanotayaroj, A. Warburton, C. P. Ward, D. R. Wardrope, A. Washbrook, P. M. Watkins, A. T. Watson, M. F. Watson, G. Watts, S. Watts, B. M. Waugh, A. F. Webb, S. Webb, M. S. Weber, S. M. Weber, S. W. Weber, S. A. Weber, J. S. Webster, A. R. Weidberg, B. Weinert, J. Weingarten, M. Weirich, C. Weiser, H. Weits, P. S. Wells, T. Wenaus, T. Wengler, S. Wenig, N. Wermes, M. D. Werner, P. Werner, M. Wessels, T. D. Weston, K. Whalen, N. L. Whallon, A. M. Wharton, A. S. White, A. White, M. J. White, R. White, D. Whiteson, B. W. Whitmore, F. J. Wickens, W. Wiedenmann, M. Wielers, C. Wiglesworth, L. A. M. Wiik-Fuchs, A. Wildauer, F. Wilk, H. G. Wilkens, H. H. Williams, S. Williams, C. Willis, S. Willocq, J. A. Wilson, I. Wingerter-Seez, E. Winkels, F. Winklmeier, O. J. Winston, B. T. Winter, M. Wittgen, M. Wobisch, T. M. H. Wolf, R. Wolff, M. W. Wolter, H. Wolters, V. W. S. Wong, N. L. Woods, S. D. Worm, B. K. Wosiek, J. Wotschack, K. W. Wozniak, M. Wu, S. L. Wu, X. Wu, Y. Wu, T. R. Wyatt, B. M. Wynne, S. Xella, Z. Xi, L. Xia, D. Xu, L. Xu, T. Xu, W. Xu, B. Yabsley, S. Yacoob, D. Yamaguchi, Y. Yamaguchi, A. Yamamoto, S. Yamamoto, T. Yamanaka, F. Yamane, M. Yamatani, T. Yamazaki, Y. Yamazaki, Z. Yan, H. Yang, H. Yang, Y. Yang, Z. Yang, W-M. Yao, Y. C. Yap, Y. Yasu, E. Yatsenko, K. H. Yau Wong, J. Ye, S. Ye, I. Yeletskikh, E. Yigitbasi, E. Yildirim, K. Yorita, K. Yoshihara, C. Young, C. J. S. Young, J. Yu, J. Yu, S. P. Y. Yuen, I. Yusuff, B. Zabinski, G. Zacharis, R. Zaidan, A. M. Zaitsev, N. Zakharchuk, J. Zalieckas, A. Zaman, S. Zambito, D. Zanzi, C. Zeitnitz, G. Zemaityte, A. Zemla, J. C. Zeng, Q. Zeng, O. Zenin, T. Ženiš, D. Zerwas, D. Zhang, D. Zhang, F. Zhang, G. Zhang, H. Zhang, J. Zhang, L. Zhang, L. Zhang, M. Zhang, P. Zhang, R. Zhang, R. Zhang, X. Zhang, Y. Zhang, Z. Zhang, X. Zhao, Y. Zhao, Z. Zhao, A. Zhemchugov, B. Zhou, C. Zhou, L. Zhou, M. Zhou, M. Zhou, N. Zhou, Y. Zhou, C. G. Zhu, H. Zhu, J. Zhu, Y. Zhu, X. Zhuang, K. Zhukov, A. Zibell, D. Zieminska, N. I. Zimine, C. Zimmermann, S. Zimmermann, Z. Zinonos, M. Zinser, M. Ziolkowski, L. Živković, G. Zobernig, A. Zoccoli, R. Zou, M. zur Nedden, L. Zwalinski, ATLAS Collaboration

**Affiliations:** 1 Department of Physics, University of Adelaide, Adelaide, Australia; 2 Physics Department, SUNY Albany, Albany, NY USA; 3 Department of Physics, University of Alberta, Edmonton, AB Canada; 4 Department of Physics, Ankara University, Ankara, Turkey; 5 Istanbul Aydin University, Istanbul, Turkey; 6 Division of Physics, TOBB University of Economics and Technology, Ankara, Turkey; 7 LAPP, CNRS/IN2P3 and Université Savoie Mont Blanc, Annecy-le-Vieux, France; 8 High Energy Physics Division, Argonne National Laboratory, Argonne, IL USA; 9 Department of Physics, University of Arizona, Tucson, AZ USA; 10 Department of Physics, The University of Texas at Arlington, Arlington, TX USA; 11 Physics Department, National and Kapodistrian University of Athens, Athens, Greece; 12 Physics Department, National Technical University of Athens, Zografou, Greece; 13 Department of Physics, The University of Texas at Austin, Austin, TX USA; 14 Institute of Physics, Azerbaijan Academy of Sciences, Baku, Azerbaijan; 15 Institut de Física d’Altes Energies (IFAE), The Barcelona Institute of Science and Technology, Barcelona, Spain; 16 Institute of Physics, University of Belgrade, Belgrade, Serbia; 17 Department for Physics and Technology, University of Bergen, Bergen, Norway; 18 Physics Division, Lawrence Berkeley National Laboratory and University of California, Berkeley, CA USA; 19 Department of Physics, Humboldt University, Berlin, Germany; 20 Albert Einstein Center for Fundamental Physics, Laboratory for High Energy Physics, University of Bern, Bern, Switzerland; 21 School of Physics and Astronomy, University of Birmingham, Birmingham, UK; 22 Department of Physics, Bogazici University, Istanbul, Turkey; 23 Department of Physics Engineering, Gaziantep University, Gaziantep, Turkey; 24 Faculty of Engineering and Natural Sciences, Istanbul Bilgi University, Istanbul, Turkey; 25 Faculty of Engineering and Natural Sciences, Bahcesehir University, Istanbul, Turkey; 26 Centro de Investigaciones, Universidad Antonio Narino, Bogotá, Colombia; 27 INFN Sezione di Bologna, Bologna, Italy; 28 Dipartimento di Fisica e Astronomia, Università di Bologna, Bologna, Italy; 29 Physikalisches Institut, University of Bonn, Bonn, Germany; 30 Department of Physics, Boston University, Boston, MA USA; 31 Department of Physics, Brandeis University, Waltham, MA USA; 32 Universidade Federal do Rio De Janeiro COPPE/EE/IF, Rio de Janeiro, Brazil; 33 Electrical Circuits Department, Federal University of Juiz de Fora (UFJF), Juiz de Fora, Brazil; 34 Federal University of Sao Joao del Rei (UFSJ), Sao Joao del Rei, Brazil; 35 Instituto de Fisica, Universidade de Sao Paulo, São Paulo, Brazil; 36 Physics Department, Brookhaven National Laboratory, Upton, NY USA; 37 Transilvania University of Brasov, Brasov, Romania; 38 Horia Hulubei National Institute of Physics and Nuclear Engineering, Bucharest, Romania; 39 Department of Physics, Alexandru Ioan Cuza University of Iasi, Iasi, Romania; 40 Physics Department, National Institute for Research and Development of Isotopic and Molecular Technologies, Cluj-Napoca, Romania; 41 University Politehnica Bucharest, Bucharest, Romania; 42 West University in Timisoara, Timisoara, Romania; 43 Departamento de Física, Universidad de Buenos Aires, Buenos Aires, Argentina; 44 Cavendish Laboratory, University of Cambridge, Cambridge, UK; 45 Department of Physics, Carleton University, Ottawa, ON Canada; 46 CERN, Geneva, Switzerland; 47 Enrico Fermi Institute, University of Chicago, Chicago, IL USA; 48 Departamento de Física, Pontificia Universidad Católica de Chile, Santiago, Chile; 49 Departamento de Física, Universidad Técnica Federico Santa María, Valparaiso, Chile; 50 Institute of High Energy Physics, Chinese Academy of Sciences, Beijing, China; 51 Department of Physics, Nanjing University, Nanjing, Jiangsu China; 52 Physics Department, Tsinghua University, Beijing, 100084 China; 53 Department of Modern Physics and State Key Laboratory of Particle Detection and Electronics, University of Science and Technology of China, Anhui, China; 54 School of Physics, Shandong University, Jinan, Shandong China; 55 Department of Physics and Astronomy, Key Laboratory for Particle Physics, Astrophysics and Cosmology, Ministry of Education, Shanghai Key Laboratory for Particle Physics and Cosmology, Shanghai Jiao Tong University, Shanghai (also at PKU-CHEP), Shanghai, China; 56 Université Clermont Auvergne, CNRS/IN2P3, LPC, Clermont-Ferrand, France; 57 Nevis Laboratory, Columbia University, Irvington, NY USA; 58 Niels Bohr Institute, University of Copenhagen, Copenhagen, Denmark; 59 INFN Gruppo Collegato di Cosenza, Laboratori Nazionali di Frascati, Frascati, Italy; 60 Dipartimento di Fisica, Università della Calabria, Rende, Italy; 61 Faculty of Physics and Applied Computer Science, AGH University of Science and Technology, Kraków, Poland; 62 Marian Smoluchowski Institute of Physics, Jagiellonian University, Kraków, Poland; 63 Institute of Nuclear Physics, Polish Academy of Sciences, Kraków, Poland; 64 Physics Department, Southern Methodist University, Dallas, TX USA; 65 Physics Department, University of Texas at Dallas, Richardson, TX USA; 66 DESY, Hamburg and Zeuthen, Germany; 67 Lehrstuhl für Experimentelle Physik IV, Technische Universität Dortmund, Dortmund, Germany; 68 Institut für Kern- und Teilchenphysik, Technische Universität Dresden, Dresden, Germany; 69 Department of Physics, Duke University, Durham, NC USA; 70 SUPA-School of Physics and Astronomy, University of Edinburgh, Edinburgh, UK; 71 INFN e Laboratori Nazionali di Frascati, Frascati, Italy; 72 Fakultät für Mathematik und Physik, Albert-Ludwigs-Universität, Freiburg, Germany; 73 Departement de Physique Nucleaire et Corpusculaire, Université de Genève, Geneva, Switzerland; 74 INFN Sezione di Genova, Genoa, Italy; 75 Dipartimento di Fisica, Università di Genova, Genoa, Italy; 76 E. Andronikashvili Institute of Physics, Iv. Javakhishvili Tbilisi State University, Tbilisi, Georgia; 77 High Energy Physics Institute, Tbilisi State University, Tbilisi, Georgia; 78 II Physikalisches Institut, Justus-Liebig-Universität Giessen, Giessen, Germany; 79 SUPA-School of Physics and Astronomy, University of Glasgow, Glasgow, UK; 80 II Physikalisches Institut, Georg-August-Universität, Göttingen, Germany; 81 Laboratoire de Physique Subatomique et de Cosmologie, Université Grenoble-Alpes, CNRS/IN2P3, Grenoble, France; 82 Laboratory for Particle Physics and Cosmology, Harvard University, Cambridge, MA USA; 83 Kirchhoff-Institut für Physik, Ruprecht-Karls-Universität Heidelberg, Heidelberg, Germany; 84 Physikalisches Institut, Ruprecht-Karls-Universität Heidelberg, Heidelberg, Germany; 85 Faculty of Applied Information Science, Hiroshima Institute of Technology, Hiroshima, Japan; 86 Department of Physics, The Chinese University of Hong Kong, Shatin, NT Hong Kong; 87 Department of Physics, The University of Hong Kong, Hong Kong, China; 88 Department of Physics, Institute for Advanced Study, The Hong Kong University of Science and Technology, Clear Water Bay, Kowloon, Hong Kong, China; 89 Department of Physics, National Tsing Hua University, Taiwan, Taiwan; 90 Department of Physics, Indiana University, Bloomington, IN USA; 91 Institut für Astro- und Teilchenphysik, Leopold-Franzens-Universität, Innsbruck, Austria; 92 University of Iowa, Iowa City, IA USA; 93 Department of Physics and Astronomy, Iowa State University, Ames, IA USA; 94 Joint Institute for Nuclear Research, JINR Dubna, Dubna, Russia; 95 KEK, High Energy Accelerator Research Organization, Tsukuba, Japan; 96 Graduate School of Science, Kobe University, Kobe, Japan; 97 Faculty of Science, Kyoto University, Kyoto, Japan; 98 Kyoto University of Education, Kyoto, Japan; 99 Research Center for Advanced Particle Physics and Department of Physics, Kyushu University, Fukuoka, Japan; 100 Instituto de Física La Plata, Universidad Nacional de La Plata and CONICET, La Plata, Argentina; 101 Physics Department, Lancaster University, Lancaster, UK; 102 INFN Sezione di Lecce, Lecce, Italy; 103 Dipartimento di Matematica e Fisica, Università del Salento, Lecce, Italy; 104 Oliver Lodge Laboratory, University of Liverpool, Liverpool, UK; 105 Department of Experimental Particle Physics, Jožef Stefan Institute and Department of Physics, University of Ljubljana, Ljubljana, Slovenia; 106 School of Physics and Astronomy, Queen Mary University of London, London, UK; 107 Department of Physics, Royal Holloway University of London, Surrey, UK; 108 Department of Physics and Astronomy, University College London, London, UK; 109 Louisiana Tech University, Ruston, LA USA; 110 Laboratoire de Physique Nucléaire et de Hautes Energies, UPMC and Université Paris-Diderot and CNRS/IN2P3, Paris, France; 111 Fysiska institutionen, Lunds universitet, Lund, Sweden; 112 Departamento de Fisica Teorica C-15, Universidad Autonoma de Madrid, Madrid, Spain; 113 Institut für Physik, Universität Mainz, Mainz, Germany; 114 School of Physics and Astronomy, University of Manchester, Manchester, UK; 115 CPPM, Aix-Marseille Université and CNRS/IN2P3, Marseille, France; 116 Department of Physics, University of Massachusetts, Amherst, MA USA; 117 Department of Physics, McGill University, Montreal, QC Canada; 118 School of Physics, University of Melbourne, Victoria, Australia; 119 Department of Physics, The University of Michigan, Ann Arbor, MI USA; 120 Department of Physics and Astronomy, Michigan State University, East Lansing, MI USA; 121 INFN Sezione di Milano, Milan, Italy; 122 Dipartimento di Fisica, Università di Milano, Milan, Italy; 123 B.I. Stepanov Institute of Physics, National Academy of Sciences of Belarus, Minsk, Republic of Belarus; 124 Research Institute for Nuclear Problems of Byelorussian State University, Minsk, Republic of Belarus; 125 Group of Particle Physics, University of Montreal, Montreal, QC Canada; 126 P.N. Lebedev Physical Institute of the Russian Academy of Sciences, Moscow, Russia; 127 Institute for Theoretical and Experimental Physics (ITEP), Moscow, Russia; 128 National Research Nuclear University MEPhI, Moscow, Russia; 129 D.V. Skobeltsyn Institute of Nuclear Physics, M.V. Lomonosov Moscow State University, Moscow, Russia; 130 Fakultät für Physik, Ludwig-Maximilians-Universität München, Munich, Germany; 131 Max-Planck-Institut für Physik (Werner-Heisenberg-Institut), Munich, Germany; 132 Nagasaki Institute of Applied Science, Nagasaki, Japan; 133 Graduate School of Science and Kobayashi-Maskawa Institute, Nagoya University, Nagoya, Japan; 134 INFN Sezione di Napoli, Naples, Italy; 135 Dipartimento di Fisica, Università di Napoli, Naples, Italy; 136 Department of Physics and Astronomy, University of New Mexico, Albuquerque, NM USA; 137 Institute for Mathematics, Astrophysics and Particle Physics, Radboud University Nijmegen/Nikhef, Nijmegen, The Netherlands; 138 Nikhef National Institute for Subatomic Physics and University of Amsterdam, Amsterdam, The Netherlands; 139 Department of Physics, Northern Illinois University, DeKalb, IL USA; 140 Budker Institute of Nuclear Physics, SB RAS, Novosibirsk, Russia; 141 Department of Physics, New York University, New York, NY USA; 142 Ohio State University, Columbus, OH USA; 143 Faculty of Science, Okayama University, Okayama, Japan; 144 Homer L. Dodge Department of Physics and Astronomy, University of Oklahoma, Norman, OK USA; 145 Department of Physics, Oklahoma State University, Stillwater, OK USA; 146 Palacký University, RCPTM, Olomouc, Czech Republic; 147 Center for High Energy Physics, University of Oregon, Eugene, OR USA; 148 LAL, Univ. Paris-Sud, CNRS/IN2P3, Université Paris-Saclay, Orsay, France; 149 Graduate School of Science, Osaka University, Osaka, Japan; 150 Department of Physics, University of Oslo, Oslo, Norway; 151 Department of Physics, Oxford University, Oxford, UK; 152 INFN Sezione di Pavia, Pavia, Italy; 153 Dipartimento di Fisica, Università di Pavia, Pavia, Italy; 154 Department of Physics, University of Pennsylvania, Philadelphia, PA USA; 155 National Research Centre “Kurchatov Institute” B.P. Konstantinov Petersburg Nuclear Physics Institute, St. Petersburg, Russia; 156 INFN Sezione di Pisa, Pisa, Italy; 157 Dipartimento di Fisica E. Fermi, Università di Pisa, Pisa, Italy; 158 Department of Physics and Astronomy, University of Pittsburgh, Pittsburgh, PA USA; 159 Laboratório de Instrumentação e Física Experimental de Partículas-LIP, Lisbon, Portugal; 160 Faculdade de Ciências, Universidade de Lisboa, Lisbon, Portugal; 161 Department of Physics, University of Coimbra, Coimbra, Portugal; 162 Centro de Física Nuclear da Universidade de Lisboa, Lisbon, Portugal; 163 Departamento de Fisica, Universidade do Minho, Braga, Portugal; 164 Departamento de Fisica Teorica y del Cosmos, Universidad de Granada, Granada, Spain; 165 Dep Fisica and CEFITEC of Faculdade de Ciencias e Tecnologia, Universidade Nova de Lisboa, Caparica, Portugal; 166 Institute of Physics, Academy of Sciences of the Czech Republic, Prague, Czech Republic; 167 Czech Technical University in Prague, Prague, Czech Republic; 168 Faculty of Mathematics and Physics, Charles University, Prague, Czech Republic; 169 State Research Center Institute for High Energy Physics (Protvino), NRC KI, Protvino, Russia; 170 Particle Physics Department, Rutherford Appleton Laboratory, Didcot, UK; 171 INFN Sezione di Roma, Rome, Italy; 172 Dipartimento di Fisica, Sapienza Università di Roma, Rome, Italy; 173 INFN Sezione di Roma Tor Vergata, Rome, Italy; 174 Dipartimento di Fisica, Università di Roma Tor Vergata, Rome, Italy; 175 INFN Sezione di Roma Tre, Rome, Italy; 176 Dipartimento di Matematica e Fisica, Università Roma Tre, Rome, Italy; 177 Faculté des Sciences Ain Chock, Réseau Universitaire de Physique des Hautes Energies-Université Hassan II, Casablanca, Morocco; 178 Centre National de l’Energie des Sciences Techniques Nucleaires, Rabat, Morocco; 179 Faculté des Sciences Semlalia, Université Cadi Ayyad, LPHEA-Marrakech, Marrakech, Morocco; 180 Faculté des Sciences, Université Mohamed Premier and LPTPM, Oujda, Morocco; 181 Faculté des Sciences, Université Mohammed V, Rabat, Morocco; 182 DSM/IRFU (Institut de Recherches sur les Lois Fondamentales de l’Univers), CEA Saclay (Commissariat à l’Energie Atomique et aux Energies Alternatives), Gif-sur-Yvette, France; 183 Santa Cruz Institute for Particle Physics, University of California Santa Cruz, Santa Cruz, CA USA; 184 Department of Physics, University of Washington, Seattle, WA USA; 185 Department of Physics and Astronomy, University of Sheffield, Sheffield, UK; 186 Department of Physics, Shinshu University, Nagano, Japan; 187 Department Physik, Universität Siegen, Siegen, Germany; 188 Department of Physics, Simon Fraser University, Burnaby, BC Canada; 189 SLAC National Accelerator Laboratory, Stanford, CA USA; 190 Faculty of Mathematics, Physics and Informatics, Comenius University, Bratislava, Slovak Republic; 191 Department of Subnuclear Physics, Institute of Experimental Physics of the Slovak Academy of Sciences, Kosice, Slovak Republic; 192 Department of Physics, University of Cape Town, Cape Town, South Africa; 193 Department of Physics, University of Johannesburg, Johannesburg, South Africa; 194 School of Physics, University of the Witwatersrand, Johannesburg, South Africa; 195 Department of Physics, Stockholm University, Stockholm, Sweden; 196 The Oskar Klein Centre, Stockholm, Sweden; 197 Physics Department, Royal Institute of Technology, Stockholm, Sweden; 198 Departments of Physics and Astronomy and Chemistry, Stony Brook University, Stony Brook, NY USA; 199 Department of Physics and Astronomy, University of Sussex, Brighton, UK; 200 School of Physics, University of Sydney, Sydney, Australia; 201 Institute of Physics, Academia Sinica, Taipei, Taiwan; 202 Department of Physics, Technion: Israel Institute of Technology, Haifa, Israel; 203 Raymond and Beverly Sackler School of Physics and Astronomy, Tel Aviv University, Tel Aviv, Israel; 204 Department of Physics, Aristotle University of Thessaloniki, Thessaloníki, Greece; 205 International Center for Elementary Particle Physics and Department of Physics, The University of Tokyo, Tokyo, Japan; 206 Graduate School of Science and Technology, Tokyo Metropolitan University, Tokyo, Japan; 207 Department of Physics, Tokyo Institute of Technology, Tokyo, Japan; 208 Tomsk State University, Tomsk, Russia; 209 Department of Physics, University of Toronto, Toronto, ON Canada; 210 INFN-TIFPA, Trento, Italy; 211 University of Trento, Trento, Italy; 212 TRIUMF, Vancouver, BC Canada; 213 Department of Physics and Astronomy, York University, Toronto, ON Canada; 214 Faculty of Pure and Applied Sciences, and Center for Integrated Research in Fundamental Science and Engineering, University of Tsukuba, Tsukuba, Japan; 215 Department of Physics and Astronomy, Tufts University, Medford, MA USA; 216 Department of Physics and Astronomy, University of California Irvine, Irvine, CA USA; 217 INFN Gruppo Collegato di Udine, Sezione di Trieste, Udine, Italy; 218 ICTP, Trieste, Italy; 219 Dipartimento di Chimica, Fisica e Ambiente, Università di Udine, Udine, Italy; 220 Department of Physics and Astronomy, University of Uppsala, Uppsala, Sweden; 221 Department of Physics, University of Illinois, Urbana, IL USA; 222 Instituto de Fisica Corpuscular (IFIC), Centro Mixto Universidad de Valencia-CSIC, Valencia, Spain; 223 Department of Physics, University of British Columbia, Vancouver, BC Canada; 224 Department of Physics and Astronomy, University of Victoria, Victoria, BC Canada; 225 Department of Physics, University of Warwick, Coventry, UK; 226 Waseda University, Tokyo, Japan; 227 Department of Particle Physics, The Weizmann Institute of Science, Rehovot, Israel; 228 Department of Physics, University of Wisconsin, Madison, WI USA; 229 Fakultät für Physik und Astronomie, Julius-Maximilians-Universität, Würzburg, Germany; 230 Fakultät für Mathematik und Naturwissenschaften, Fachgruppe Physik, Bergische Universität Wuppertal, Wuppertal, Germany; 231 Department of Physics, Yale University, New Haven, CT USA; 232 Yerevan Physics Institute, Yerevan, Armenia; 233 Centre de Calcul de l’Institut National de Physique Nucléaire et de Physique des Particules (IN2P3), Villeurbanne, France; 234 Academia Sinica Grid Computing, Institute of Physics, Academia Sinica, Taipei, Taiwan; 235 CERN, 1211 Geneva 23, Switzerland

## Abstract

This paper presents a measurement of the polarisation of $\tau$ leptons produced in $Z/\gamma ^{*}\rightarrow \tau \tau$ decays which is performed with a dataset of proton—proton collisions at $\sqrt{s}=8$ TeV, corresponding to an integrated luminosity of 20.2 fb$^{-1}$ recorded with the ATLAS detector at the LHC in 2012. The $Z/\gamma ^{*}\rightarrow \tau \tau$ decays are reconstructed from a hadronically decaying $\tau$ lepton with a single charged particle in the final state, accompanied by a $\tau$ lepton that decays leptonically. The $\tau$ polarisation is inferred from the relative fraction of energy carried by charged and neutral hadrons in the hadronic $\tau$ decays. The polarisation is measured in a fiducial region that corresponds to the kinematic region accessible to this analysis. The $\tau$ polarisation extracted over the full phase space within the $Z/\gamma ^{*}$ mass range of 66 $< m_{Z/\gamma ^{*}} <$ 116 GeV is found to be $P_{\tau } =-0.14 \pm 0.02 (\text {stat}) \pm 0.04 (\text {syst})$. It is in agreement with the Standard Model prediction of $P_{\tau } =-0.1517 \pm 0.0019$, which is obtained from the ALPGEN event generator interfaced with the PYTHIA 6 parton shower modelling and the TAUOLA $\tau$ decay library.

## Introduction

The $\tau$ lepton plays an important role in the physics programme of the Large Hadron Collider (LHC). It is used to identify and measure electroweak and top quark production processes as well as in searches for new physics beyond the Standard Model. Since the $\tau$ leptons decay before exiting the ATLAS detector volume, their polarisation can be measured.

The $\tau$ polarisation, $P_{\tau }$, is the asymmetry of the cross-section for positive ($\sigma _+$) or negative ($\sigma _-$) helicity $\tau$ lepton production, defined by:

1 $$\begin{aligned} P_\tau = \frac{\sigma _+ - \sigma _-}{\sigma _+ + \sigma _-} \end{aligned}$$

for the $\tau ^{-}$ lepton. It is a measure of the degree of parity violation in the interaction producing the $\tau$ leptons and therefore it provides insight into the nature of its Lorentz structure. The positive (negative) helicity states and right-handed (left-handed) chiral states coincide in the relativistic limit assumed here.^1^ Due to nearly exact $CP$ invariance in $\tau$ decays, the kinematic distributions for left-handed (right-handed) $\tau ^{+}$ follow those of right-handed (left-handed) $\tau ^{-}$. Therefore, in this paper only one of the equivalent *CP* states is mentioned at a time with the other being implicitly assumed. Any possible differences are negligible for the measurement described in this paper.

The $\tau$ polarisation in $Z\rightarrow \tau \tau$ decays was first measured at LEP in electron–positron annihilation events at the *Z* boson pole. The experiments at LEP published the $P_\tau$ spectrum as a function of the angle between the directions of the $\tau ^{-}$ lepton and the $e^{-}$ beam [1]. The most precise value of the average $\tau$ polarisation was obtained in the combination of LEP results and presented in terms of the $\tau$ production asymmetry, $A_{\tau }$, which, by convention, has reversed sign with respect to the polarisation and contains small (${\mathcal {O}}(0.005)$) corrections for the interference between the *Z* boson and photon propagators as well as for the pure photon contribution. The asymmetry value obtained in the combination is $A_{\tau } = 0.1439 \pm 0.0043$ [1].

The measurement presented in this paper provides a complementary constraint on the $\tau$ polarisation in decays of $Z/\gamma ^*$ that are produced via a *qqZ* vertex in proton–proton collisions as the quark-electroweak couplings are involved. It is performed by analysing $Z/\gamma ^{*}\rightarrow \tau \tau$ decays in which one $\tau$ decays leptonically ($\tau \rightarrow e/\mu +\nu \nu$) and the other hadronically ($\tau \rightarrow \text {hadron(s)}+\nu$). The leptonic decay is utilised to trigger, select, and identify $Z/\gamma ^{*}\rightarrow \tau \tau$ candidate events, while the hadronic decay serves as a spin analyser. The $qq\rightarrow Z\rightarrow \tau \tau$ signal has been observed before by the ATLAS, CMS and LHCb collaborations [6–8]. Due to the abundance of background processes, strict requirements are applied to select a sufficiently pure sample of $Z/\gamma ^{*}\rightarrow \tau \tau$ decays from the proton–proton collision data. Further requirements are dictated by the detector acceptance. The overall acceptance is larger for $Z/\gamma ^{*}\rightarrow \tau \tau$ decays with left-handed $\tau ^{-}$. To provide a result that is close to the polarisation directly observed in the selected signal region, the $\tau$ polarisation is measured in a fiducial region, which is defined at stable-particle level and very similar to the selected signal region. The polarisation is predicted by using simulated event samples produced with the Alpgen [2] event generator interfaced with the Pythia6 [3] parton shower and hadronisation model. The $\tau$ lepton decay and spin effects are simulated with the Tauola [4] decay library using $\sin ^2\theta _\text {W}^\text {eff}=0.23147$ in the electroweak leading-order (LO) matrix element to simulate polarisation and spin correlations in the Tauola Universal Interface [5]. The prediction in the fiducial region is $P_{\tau } = -0.270\pm 0.006$.

The principal result presented in this paper is a measurement of the $\tau$ polarisation inside the $Z/\gamma ^{*}$ mass range of 66 $< m_{Z/\gamma ^{*}}<$ 116 GeV. Away from the *Z* boson mass peak, the degree of polarisation varies with $m_{Z/\gamma ^{*}}$ and is determined by the interference between *Z* boson- and photon-mediated amplitudes. An inclusive measurement over a mass range around the *Z* boson pole is performed here, because the contributions slightly above and below the *Z* boson pole cannot be separated accurately. The $Z/\gamma ^{*}$ interference has approximately the opposite effect on the polarisation below and above the *Z* boson pole. Therefore, and because the on-pole cross-section is dominant, the polarisation inside the mass-selected region of 66 $< m_{Z/\gamma ^{*}}<$ 116 GeV is close to $P_\tau$ at $\sqrt{s}=m_Z$. The prediction by the Alpgen event generator interfaced with the Pythia6 parton shower and hadronisation model and Tauola library for $\tau$ decays is $P_\tau ~=-0.1517\pm 0.0014\;(\text {stat})\pm 0.0013\;(\text {syst})$. This is different from the $P_{\tau }$ value in fiducial region because some of the event selection requirements, such as transverse momenta thresholds, prefer one $\tau$ helicity state over another. For the extrapolation from the selected signal region to the full phase space inside the $Z/\gamma ^{*}$ mass range, the $\tau \tau$ contribution is assumed to originate from $Z/\gamma ^{*}\rightarrow \tau \tau$ decays. In particular, the spin correlations of the two $\tau$ leptons are assumed to be those for unit-spin intermediate states. The $\tau$ decays are assumed to follow the Standard Model expectations.

The first $\tau$ polarisation measurement at ATLAS was performed in $W\rightarrow \tau \nu$ decays in proton–proton collisions at the centre-of-mass energy of $\sqrt{s}=7$ TeV recorded in 2010 [9]. The concept to extract the polarisation from a template fit to a polarisation sensitive observable is retained from that analysis. To exploit the larger dataset collected at $\sqrt{s}=8$ TeV, refined experimental techniques for $\tau$ polarisation measurements at hadron colliders are utilised for the measurement presented in this paper. In particular, the impact of systematic uncertainties in the modelling of the polarisation observable for signal events and the significant backgrounds are estimated more thoroughly, because they are more important in the current measurement using a larger dataset. These techniques may serve as a foundation for future polarisation measurements in decays of the Higgs boson or $\tau \tau$ final states with high invariant mass. A good understanding of $\tau$ polarisation in *Z* boson decays is indispensable for these measurements. Moreover, the polarisation itself provides a potential discriminant in Standard Model Higgs boson selection and searches for physics beyond the Standard Model. In particular it may help to distinguish decays of heavy particles where the same final states involving $\tau$ leptons are predicted but with different helicity configurations, such as for separating *Z* and *H* or *A* bosons or for distinguishing *W* and $H^{\pm }$ bosons.

This paper is structured as follows. In Sect. 2 an overview of the ATLAS detector is presented. The event samples, which were recorded by ATLAS or simulated using the ATLAS simulation framework, are introduced in Sect. 3. The reconstruction and definition of physics objects is documented in Sect. 4. Section 5 describes the selected signal region and the prediction of the polarisation in the fiducial region and in the mass-selected region. The $\tau$ polarisation observable is introduced in Sect. 6. The estimation of the background contributions in the selected signal region is documented in Sect. 7. Section 8 describes the estimation of the experimental and theory systematic uncertainties. A description of the fit model used to extract the $\tau$ polarisation is given in Sect. 9. The results of the measurement are shown in Sect. 10, followed by conclusions in Sect. 11.

## ATLAS detector

The ATLAS experiment [10] at the LHC is a multi-purpose particle detector with a forward-backward symmetric cylindrical geometry and a near $4\pi$ coverage in solid angle.^2^ It consists of an inner tracking detector surrounded by a thin superconducting solenoid providing a 2 T axial magnetic field, electromagnetic and hadronic calorimeters, and a muon spectrometer. The inner tracking detector covers the pseudorapidity range $|\eta | < 2.5$. It consists of silicon pixel, silicon microstrip, and transition radiation tracking detectors. Lead/liquid-argon (LAr) sampling calorimeters provide electromagnetic (EM) energy measurements with high granularity. A hadronic (steel/scintillator-tile) calorimeter covers the central pseudorapidity range ($|\eta | < 1.7$). The endcap and forward regions are instrumented with LAr calorimeters for both the EM and hadronic energy measurements up to $|\eta | = 4.9$. The muon spectrometer surrounds the calorimeters and features three large air-core toroid superconducting magnets with eight coils each. The field integral of the toroids ranges from 2.0 to $6.0\hbox { T}\cdot \hbox {m}$ across most of the detector. It includes a system of precision tracking chambers and fast detectors for triggering. A three-level trigger system is used to select events [11]. The first-level trigger is implemented in hardware and uses a subset of the detector information to reduce the accepted rate to at most 75 kHz. This is followed by two software-based trigger levels that together reduce the accepted event rate to 400 Hz on average depending on the data-taking conditions during 2012.

## Data and simulated event samples

The data sample was recorded by ATLAS in proton–proton collisions provided by the LHC at a centre-of-mass energy of $\sqrt{s}=8$ TeV in 2012. The integrated luminosity of the sample is ${\mathcal {L}}=20.2$ fb$^{-1}$ after beam and data quality requirements are satisfied. Candidate events are selected with four triggers, a single-muon or single-electron trigger requiring an isolated muon or electron with transverse momentum $p_{\text {T}}\,>24$ GeV complemented by higher-threshold ($p_{\text {T}}\,>60$ GeV for electrons, $p_{\text {T}}\,>36$ GeV for muons) triggers without isolation requirements. The accepted events must also contain at least one reconstructed primary vertex with more than three tracks with $p_{\text {T}}\,>400$ MeV each. If more than one such vertex is present, that with the highest sum of the squared transverse momenta of all associated tracks is chosen as the primary vertex.

The expected signal as well as several background processes are modelled using samples of simulated events. Signal ($Z/\gamma ^{*}\rightarrow \tau \tau$) + jets events were generated with boson masses $m_{Z/\gamma ^{*}}>60$ GeV with the Alpgen event generator interfaced with the Pythia6 fragmentation, hadronisation and underlying event (UE) modelling. The Alpgen event generator was used with default electroweak parameters [2]. The CTEQ6L1 [12] parton distribution function (PDF) set and a set of tuned parameters called the Perugia2011C tune [13] were used. QED radiation was simulated by the Photos [14] algorithm. The information about the $\tau$ helicity state was not stored at the generation step for the ($Z/\gamma ^{*}\rightarrow \tau \tau$) + jets process. The spin polarisation and correlations were therefore simulated using Tauola Universal Interface [5] as expected from the electroweak lowest-order matrix element for the $Z/\gamma ^{*}\rightarrow \tau \tau$ production process, with $\sin ^2\theta _\text {W}^\text {eff}=0.23147$. The $\tau$ decays were simulated using the Tauola decay library [4]. The helicities of $\tau$ leptons generated by the Tauola algorithm were not stored so that the helicity is reconstructed in the generated signal samples with the TauSpinner [15] package associated with the Tauola decay library. The TauSpinner algorithm assigns the helicity of $\tau$ leptons randomly based on probabilities derived from the kinematic configuration of the $\tau$ decays. The signal sample is thereby split into events with left-handed $\tau ^{-}$ (and right-handed $\tau ^{+}$) and those with right-handed $\tau ^{-}$ (and left-handed $\tau ^{+}$). The TauSpinner algorithm averages over incoming parton flavours and four-momenta whereas the Tauola algorithm directly accesses the incoming partons in each event. The average over initial parton states is performed using the MRSTMCal PDF set [16] in this analysis. Spin correlations as expected in $Z/\gamma ^{*}\rightarrow \tau \tau$ decays are assumed. The TauSpinner package was extensively tested and validated by its authors [15, 17, 18] and used in several measurements [19, 20].

For studies of systematic uncertainties, an auxiliary sample of $Z/\gamma ^{*}\rightarrow \tau \tau$ events was produced using the Pythia8 [21] event generator with the CTEQ6L1 PDF set and AU2 [22] tune for the UE. In this case the Pythia8 event generator was used to model both the production process and decays including those of $\tau$ leptons. Further auxiliary signal samples were produced with the Powheg [23–25] event generator interfaced with the Pythia8 parton shower simulation using the CT10 PDF set [26] and with the Alpgen event generator interfaced with the Herwig/Jimmy [27, 28] hadronisation and UE modelling. Only stable-particle-level information is used in the auxiliary samples.

Background samples of simulated $(W\rightarrow e\nu )$ + jets, $(W\rightarrow \mu \nu )$ + jets, $(W\rightarrow \tau \nu )$ + jets, $(Z/\gamma ^{*}\rightarrow ee)$ + jets, and $(Z/\gamma ^{*}\rightarrow \mu \mu )$ + jets events were generated using the Alpgen event generator interfaced with the Pythia6 hadronisation modelling and with the same settings as for the signal $Z \rightarrow \tau \tau$ sample. For these samples, LO matrix elements were calculated for up to five additional partons. The resulting predictions were scaled such that the total cross-sections match the respective inclusive next-to-next-to-leading-order (NNLO) predictions [29]. A sample of top pair production was generated using the Powheg [23–25] event generator interfaced with the Pythia6 hadronisation modelling and with the CT10 [26] PDF set. The $t\bar{t}$ cross-section was calculated at NNLO+NNLL (next-to-next-to-leading-logarithm) [24]. In this analysis all simulated event samples receive data-driven corrections to the normalisation predicted by the aforementioned cross-sections with the exception of the $t\bar{t}$ background. The list of simulated event samples used in this analysis is given in Table 1.

**Table 1:** Simulated event samples used in the analysis. The table lists the sample, the event generator, the PDF set, and the underlying-event tune

Sample	Event generator	PDF	UE tune
$(Z/\gamma ^{*}\rightarrow \tau \tau )$ + jets	Alpgen 2.14 [2] + Pythia6.427 [3]	CTEQ6L1 [12]	Perugia2011C [13]
$(Z/\gamma ^{*}\rightarrow \tau \tau )$ + jets	Pythia 8.160 [21]	CTEQ6L1	AU2 [22]
$(Z/\gamma ^{*}\rightarrow \tau \tau )$ + jets	Powheg r1556 [23–25] + Pythia 8.160	CT10 [26]	AUET2 [30]
$(Z/\gamma ^{*}\rightarrow \tau \tau )$ + jets	Alpgen 2.14 + Herwig 6.5/Jimmy 4.3 [27, 28]	CTEQ6L1	Perugia2011C
Top pairs + jets	Powheg r2129 + Pythia 6.426	CT10	AUET2
$(W\rightarrow e\nu )$ + jets	Alpgen 2.14 + Pythia 6.427	CTEQ6L1	Perugia2011C
$(W\rightarrow \mu \nu )$ + jets	Alpgen 2.14 + Pythia 6.427	CTEQ6L1	Perugia2011C
$(W\rightarrow \tau \nu )$ + jets	Alpgen 2.14 + Pythia 6.427	CTEQ6L1	Perugia2011C
$(Z/\gamma ^{*}\rightarrow ee)$ + jets	Alpgen 2.14 + Pythia 6.427	CTEQ6L1	Perugia2011C
$(Z/\gamma ^{*}\rightarrow \mu \mu )$ + jets	Alpgen 2.14 + Pythia 6.427	CTEQ6L1	Perugia2011C

The simulated $Z/\gamma ^{*}$ boson decays (in both the signal and background processes) are reweighted such that the simulated $p_{\text {T}}\,$ spectrum of the $Z/\gamma ^{*}$ bosons matches the observed $p_{\text {T}}\,$ spectrum in data, as done in Ref. [31], using $Z/\gamma ^{*}\rightarrow \mu \mu$ events. The response of the ATLAS detector was simulated [32] using Geant4 [33]. Simulated events were overlaid with additional minimum-bias events generated with the Pythia8 event generator to account for the effect of multiple interactions occurring in the same and neighbouring bunch crossings (pile-up). The simulated events were re-weighted such that the distribution of the average number of pile-up interactions per bunch crossing matches the observed spectrum in data. Finally, the simulated events were processed through the same reconstruction algorithms as the data.

## Event reconstruction and object definitions

Electrons are reconstructed from energy clusters in the calorimeter which have a matching track in the inner detector. Electron candidates are considered if they satisfy ‘loose’ identification criteria [34] and the requirements of $p_{\text {T}}\,>15$ GeV and $|\eta | < 2.47$.

Muon candidates are reconstructed from associated tracks in the inner detector and the muon spectrometer. They are required to satisfy ‘loose’ [35] identification criteria as well as the requirements of $p_{\text {T}}\,>10$ GeV and $|\eta | < 2.5$. The electron and muon (lepton) candidates that pass the aforementioned requirements are in the following referred to as preselected.

In order to be selected, lepton candidates are required to have $p_\text {T,lepton}>26$ GeV and to pass stricter identification requirements. Specifically, electron candidates must satisfy ‘tight’ [34] identification criteria and lie outside the calorimeter transition region of $1.37<|\eta |<1.52$. Muon candidates are required to have a combined track [35] in the inner detector and muon spectrometer. Additionally, isolation requirements in the inner detector and calorimeter are applied to both the electrons and muons. The fraction of the momentum carried by tracks other than the identified lepton track inside a cone of size $\Delta R=0.4$ around the lepton track must be less than 6%. Similarly, after correcting for pile-up, the fraction of the transverse energy reconstructed in a cone of size $\Delta R=0.2$ around the lepton axis but not associated with the lepton candidate must not exceed 6% of the lepton’s transverse energy.

Jets are reconstructed [36] with the anti-$k_{t}$ algorithm [37] with a radius parameter $R=0.4$ using topological clusters of energy deposits in the electromagnetic and hadronic calorimeters within $|\eta |<4.5$ with a local hadronic calibration [38]. In this analysis, jets with $p_{\mathrm {T}} > 20$ GeV are used in the calculation of missing transverse momentum. Here, jets with $|\eta |<2.4$ and $p_{\text {T}}\,<50$ GeV must meet additional criteria designed to select jets from the hard-scatter interaction and reject those originating from pile-up: among the tracks associated with the jet, those originating from the primary vertex must contribute at least 50% of the sum of the scalar $p_{\text {T}}$ of all those tracks [39]. In this analysis, no selection is made on the number of jets.

The reconstruction of $\tau$ candidates is based on the visible decay products of hadronically decaying $\tau$ leptons ($\tau _\text {had}$ with visible component $\tau _{\text {had}-\text {vis}}$). These candidates are seeded by jets reconstructed with transverse momentum above 10 GeV. At this stage of the analysis $\tau _\text {had}$ candidates are required to have reconstructed $p_{\text {T},\tau _{\text {had}-\text {vis}}}>20$ GeV and $|\eta |<2.47$, to have exactly one or three charged-particle tracks, to be identified with ‘medium’ identification criteria [40], and to have reconstructed electric charge of $\pm 1$. The $\tau _\text {had}$ energy scale is determined from simulated event samples and accounts for the mixture of hadrons typical of $\tau _\text {had}$ decays as well as contributions from the UE, pile-up, and energy outside of the $\tau _{\text {had}-\text {vis}}$ cone [40]. A ‘medium’ electron veto as well as a muon veto are applied to reject electrons and muons that are reconstructed as $\tau _\text {had}$ candidates [40].

Objects that are reconstructed in geometrically overlapping regions, given by a cone of size $\Delta R=0.2$, are identified with the above definitions with the following precedence: preselected muon, preselected electron, $\tau _\text {had}$ candidate, and jet. For the purpose of removing overlaps between muons and $\tau _\text {had}$ candidates, the $p_{\text {T}}$ threshold for muon candidates is reduced to 2 GeV.

The missing transverse momentum $(E_{\text {T}}^{\text {miss}})$ is calculated as the modulus of the negative vectorial sum of the $\varvec{p}_\mathbf{T}$ of all fully reconstructed and calibrated physics objects in the event, as well as a term for the remaining activity in the calorimeter [41]. Here, preselected leptons are included in the sum.

## Event selection

Selection criteria are applied to obtain a sample enhanced in $Z/\gamma ^{*}\rightarrow \tau \tau$ events where one of the $\tau$ leptons decays leptonically ($\tau _\text {lep}$) and the other hadronically. The $\tau _\text {had}$ candidate is required to have exactly one charged-particle track (single-prong). Events are categorised into channels by the lepton flavour (electron or muon), which are referred to as $\tau _e\textendash \tau _\text {had}$ and $\tau _\mu \textendash \tau _\text {had}$ channels. The kinematic requirements on electrons and muons are similar and, therefore, the event selections that define the two selected signal regions are described in parallel.

Exactly one $\tau _\text {had}$ candidate and exactly one lepton that fulfil the respective selection criteria and that have opposite-sign electric charges are required. Two selection requirements are implemented to reduce the significant background that arises from *W*+jets production in which a lepton is reconstructed correctly and a jet is misidentified as a $\tau _\text {had}$ candidate. The transverse mass, $m_{\mathrm {T}}$, built from the lepton and missing transverse momenta, is defined as

$$\begin{aligned} m_{\mathrm {T}} = \sqrt{2~p_{\mathrm {T,lepton}}~E_{\text {T}}^{\text {miss}} ~\left( 1-\mathrm {cos}\left( \Delta \phi \left( \mathrm {lepton},E_{\text {T}}^{\text {miss}} \right) \right) \right) } \end{aligned}$$

and is required to satisfy $m_{\mathrm {T}}<30$ GeV. The sum of the azimuthal angular separation between the $\tau _\text {had}$ candidate and the $E_{\text {T}}^{\text {miss}}$ directions, and the lepton and the $E_{\text {T}}^{\text {miss}}$ directions,

$$\begin{aligned} \sum \Delta \phi = \Delta \phi \left( \tau _{\text {had}-\text {vis}},E_{\text {T}}^{\text {miss}} \right) +\Delta \phi \left( \mathrm {lepton},E_{\text {T}}^{\text {miss}} \right) \end{aligned}$$

is required to satisfy $\sum \Delta \phi < 3.5$. This requirement suppresses event topologies in which the $E_{\text {T}}^{\text {miss}}$ lies outside of the angle spanned by the $\tau$ candidate and the lepton, which are common for *W*+jets processes and rare for signal events. In addition, the visible mass of the $\tau _\text {had}$ candidate and lepton, $m_\mathrm {vis} = m(\tau _{\text {had}-\text {vis}},\text {lepton})$, is required to satisfy 40 $< m_{\mathrm {vis}}<85$ GeV to further reduce backgrounds, notably the non-signal $Z/\gamma ^{*}$+jets background in which the $Z/\gamma ^{*}$ boson decays to electron or muon pairs. For signal events around the *Z* boson pole that pass the previous requirements, the $m_\mathrm {vis}$ distribution is centred at about 66 GeV and has a width of about 10 GeV. This is insufficient for separating $Z/\gamma ^{*}\rightarrow \tau \tau$ decays on and off the *Z* boson pole. The selection criteria described above define the selected signal region of this analysis.

Some of the object and event selection requirements have different acceptances for signal decays with one specific $\tau ^{-}$ helicity state: the $p_{\mathrm {T,lepton}}$ requirement is about twice as efficient for $Z/\gamma ^{*}\rightarrow \tau \tau$ events with leptonically decaying left-handed $\tau ^{-}$ leptons as for those with leptonically decaying right-handed $\tau ^{-}$ leptons. Here, the polarisation of the $\tau _\text {had}$ is affected due to spin correlations resulting from angular momentum conservation in $Z/\gamma ^{*}\rightarrow \tau \tau$ decays. This is partially counteracted by the $p_{\mathrm {T},\tau _{\text {had}-\text {vis}}}$ and $m_{\mathrm {T}}$ requirements. These biases result from dependencies of the $\tau$ lepton momentum share carried by neutrinos on the helicity state and the respective decay modes. The size of this effect may be different for possible unexpected contributions from physics processes other than from intermediate states with unit spin decaying to $\tau$ pairs. Hence the polarisation is also measured in a fiducial region which is defined with stable-particle-level quantities (see Table 2). It corresponds very closely to the selected signal region. For the extraction of the $\tau$ polarisation in this region, the simulated signal sample is split into three components:

- Events inside the fiducial region with left-handed $\tau ^{-}$ leptons,
- Events inside the fiducial region with right-handed $\tau ^{-}$ leptons,
- Events outside the fiducial region.

About 80% of the events in the selected signal region originate from the fiducial region. Most of the remaining events fail the $m_\mathrm {T}$, $p_{\mathrm {T},\tau _{\text {had}-\text {vis}}}$ or $p_{\mathrm {T,lepton}}$ requirements on stableparticle level but pass them at reconstructed-detector level.

**Table 2:** Definition of fiducial region for $Z/\gamma ^{*}\rightarrow \tau \tau$ decays. The requirements are applied at stable-particle level. Here, the $E_{\text {T}}^{\text {miss}}$ is calculated from the momenta of the neutrinos that originate from the $Z/\gamma ^{*}\rightarrow \tau \tau$ decays

One $\tau _\text {lep}$ decay	One single-prong $\tau _\text {had}$ decay
$p_{\mathrm {T,lepton}}>26$ GeV	$p_{\mathrm {T},\tau _{\text {had}-\text {vis}}}>20$ GeV
$|\eta _{e}|<2.47$ and not $1.37<|\eta _{e}|<1.52$ or $|\eta _\mu | < 2.5$	$|\eta _{\tau _{\text {had}-\text {vis}}}|<2.47$
$m_{\mathrm {T}}<30$ GeV	40 $< m_{\mathrm {vis}}<85$ GeV

For the extraction of the $\tau$ polarisation in $Z/\gamma ^{*}\rightarrow \tau \tau$ decays inside the mass-selected region of 66 $<m_{Z/\gamma ^{*}}<$ 116 GeV, the signal sample is split into these components:

- Events with $m_{Z/\gamma ^{*}}$ inside the mass-selected region with left-handed $\tau ^{-}$ leptons,
- Events with $m_{Z/\gamma ^{*}}$ inside the mass-selected region with right-handed $\tau ^{-}$ leptons,
- Events with $m_{Z/\gamma ^{*}}$ outside the mass-selected region,

where the mass-selected region is defined at stable-particle level. About 98% of the simulated $Z/\gamma ^{*}\rightarrow \tau \tau$ events in the selected signal region originate from the mass-selected region.

The $\tau$ polarisation is measured using the $\tau _\text {had}$ decay as a spin analyser, and without utilising spin correlations of the two $\tau$ leptons. Therefore, the polarisation measurement in the fiducial region does not strongly rely on the prediction of the $\tau$ spin correlations. The most important exception is that the contribution of $Z/\gamma ^{*}\rightarrow \tau \tau$ events which are outside the fiducial region but which fall inside the selected signal region is taken from simulation. In contrast, the polarisation measurement in the mass-selected region relies on the prediction of the spin correlations when extrapolating to the full phase space and is therefore more model-dependent. Because of that, the interpretation of the measurement in the mass-selected region is largely model-dependent, if an anomalous polarisation value is measured.

The theoretical prediction of the $\tau$ polarisation in the mass-selected region of 66 $< m_{Z/\gamma ^{*}}<$ 116 GeV is obtained by performing a fit to the distribution of the momentum fraction, *x*, carried by the $\pi ^{\pm }$ at stable-particle level in $\tau ^\pm \rightarrow \pi ^\pm \nu$ decays for events inside the mass-selected region. Specifically, this distribution follows $f(x) = 1 + P_{\tau }(2x-1)$ as described in Ref. [42]. The resulting prediction is $P_\tau ~=-0.1517\pm 0.0014\;(\text {stat})\pm 0.0013\;(\text {syst})$. It is unaffected by TauSpinner and MC-related systematic uncertainties and the quoted uncertainty results from the choice of shower model simulation and PDFs. Since the *x* distribution is altered by the fiducial region selection, the polarisation in the fiducial region can only be predicted from the numbers of events in which the $\tau ^{-}$ is classified as left- and right-handed by TauSpinner. This method is affected by TauSpinner systematic uncertainties, so the prediction of the polarisation in the fiducial region is less accurate than that of the polarisation in the mass-selected region. A predicted value of $P_{\tau } =-0.270\pm 0.006$ is obtained. Details of the estimation of particular systematic uncertainties are given in Sect. 8.2.

## Observable for $\tau$ polarisation

The helicity of the $\tau$ lepton manifests itself in the kinematic distributions of its decay products.

The $\tau$ decay mode exhibiting the highest sensitivity to the $\tau$ polarisation is $\tau ^{\pm } \rightarrow h^{\pm } \nu$, where $h^{\pm }$ denotes $\pi ^{\pm }$ or $K^{\pm }$ (branching ratio, ${\mathcal {B}}$, $\simeq 11.5\%$ [43]). The branching ratio of the decay mode involving a $\pi ^{\pm }$ exceeds that of the mode involving a $K^{\pm }$ by more than an order of magnitude. This also holds for the $\tau$ decay modes described below. In the $\tau$ rest frame, the neutrino (always left-handed) is preferentially emitted opposite to the $\tau ^{-}$ spin orientation.

The angle $\theta$ between the $\tau$ flight direction in the laboratory frame and $\pi ^{\pm }$ flight direction in the $\tau$ rest frame is the primary observable sensitive to $\tau$ polarisation. It cannot be measured directly at hadron colliders because insufficient information about the initial state is available. However, $\theta$ affects the momentum fraction carried by the $h^{\pm }$ resulting in a larger acceptance for right-handed than for left-handed $\tau ^{-}$ in $\tau ^{-}\rightarrow h^{-} \nu$ decays.

Another $\tau$ decay mode, $\tau ^{\pm } \rightarrow h^{\pm } \pi ^0 \nu$ (${\mathcal {B}}\simeq 25.9\%$ [43]), plays an important role in the polarisation measurement. It offers the kinematic simplicity of a two-body decay, since it goes mostly through sequential decays $\tau ^{\pm } \rightarrow \rho ^{\pm } \nu$, $\rho ^{\pm } \rightarrow \pi ^{\pm } \pi ^0$, but the sensitivity to the angle between the $\tau$ direction of flight and $\pi ^{\pm }$ is lower, due to the mixing of longitudinally and transversely polarised $\rho ^{\pm }$ vector mesons. The products of the $\rho ^{\pm } \rightarrow \pi ^{\pm } \pi ^0$ decay are experimentally accessible and their angular distributions as well as their energies depend on the helicity of the vector meson.

The angle between the direction of flight of the $\rho ^{\pm }$ meson and $\pi ^{\pm }$ in the $\rho ^{\pm }$ rest frame is related to the energy–sharing between the $\pi ^{\pm }$ and the $\pi ^0$ and is sensitive to the $\tau$ helicity. An asymmetry of energies carried by the charged and neutral pions and measured in the laboratory frame is defined as:

2 $$\begin{aligned} \Upsilon _{\mathrm {theory}} = \frac{E_{\pi ^{\pm }} - E_{\pi ^0}}{E_{\pi ^{\pm }} + E_{\pi ^0}}. \end{aligned}$$

This asymmetry carries high sensitivity to polarisation and was effective in measuring the $\tau$ polarisation in the decay $W\rightarrow \tau \nu$ [9].

The other decay modes considered are the modes with more neutral pions ($\tau ^{\pm } \rightarrow h^{\pm } N\pi ^0 \nu$, $N\ge 2$), and decay modes with three charged mesons, where two tracks are lost, and a small admixture of other modes. In this class of decay modes the dominant mode is $\tau ^{\pm } \rightarrow h^{\pm } 2\pi ^0 \nu$, with ${\mathcal {B}}\simeq 9.3\%$ [43]. It has more complicated kinematics than $\tau ^{\pm } \rightarrow h^{\pm } \pi ^0 \nu$, but it nonetheless contributes to the polarisation sensitivity. The contributions from other channels are small. For example the branching ratio of $\tau ^{\pm } \rightarrow h^{\pm } 3\pi ^0 \nu$ is only $\simeq 1\%$ [43].

The asymmetry defined in Eq. (2) is approximated using the experimental observables. In this approach the $p_{\text {T}}\,$ of a single track associated with the $\tau _\text {had}$ candidate replaces the energy of the $\pi ^{\pm }$. Since the energies of neutral pions are not measured directly, the difference between the $\tau$ lepton visible $E_\text {T}$, defined below, and the track $p_{\text {T}}\,$ is used in place of the $\pi ^0$ energy. As the minimum $\tau _\text {had}$ $p_{\text {T}}\,$ required is 20 GeV, the $\tau$ leptons are relativistic enough to use this approximation. The visible $E_\text {T}$ of $\tau _\text {had}$ candidates is reconstructed using the energy deposit in the calorimeter [40]. Therefore, the charged asymmetry is given by:

3 $$\begin{aligned} \Upsilon = \frac{E_{\text {T}} ^{\pi ^{\pm }} - E_{\text {T}} ^{h^0}}{E_{\text {T}} ^{\tau _{\text {had}-\text {vis}}}} = 2\frac{p_{\text {T}}\,^{\mathrm {track}}}{E_{\text {T}} ^{\tau _{\text {had}-\text {vis}}}} - 1, \end{aligned}$$

where $h^0$ denotes neutral particles produced in the $\tau$ decay, which are mostly neutral pions.

**Fig. 1:**
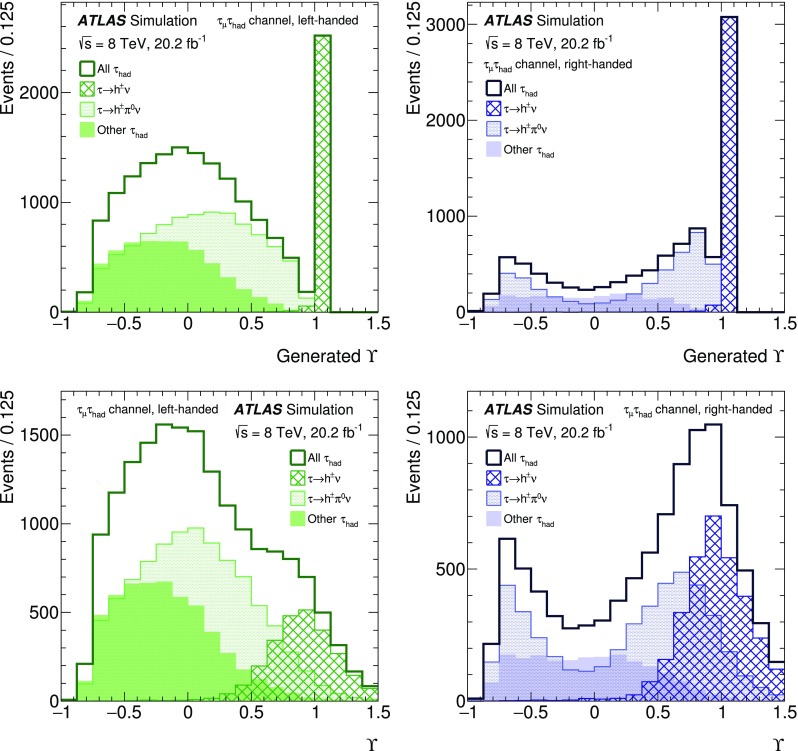
Charged asymmetry distributions as defined in Eq. (3) for left-handed (left) and right-handed (right) single-prong reconstructed $\tau _\text {had}$ leptons in simulated $Z/\gamma ^{*}\rightarrow \tau \tau$ decays after the full event selection in the $\tau _\mu \textendash \tau _\text {had}$ channel. The charged asymmetry is calculated from stable-particle level (top) and reconstructed-detector-level quantities. In addition to the inclusive distributions, the constituent distributions corresponding to generated $\tau$ leptons that decay in the $\tau \rightarrow h^{\pm }\nu$ and $\tau \rightarrow h^{\pm }\pi ^0\nu$ ($h^{\pm }$ denotes $\pi ^{\pm }$ or $K^{\pm }$) modes are overlaid, as well as that of the remaining decay modes. The latter mainly consist of $\tau \rightarrow h^{\pm }N\pi ^0\nu$ decays, where $N \ge 2$. The analysis does not, however, distinguish between the decay modes. The distributions are normalised according to their respective cross-sections. Here, the polarisation is taken from the simulation

The shapes of $\Upsilon$ distributions for the left-handed and right-handed reconstructed single-prong $\tau$ candidates obtained from simulation after the full event selection are shown in Fig. 1.

The $\Upsilon$ spectra include effects that originate from the acceptance, object reconstruction, and efficiencies as well as the event selection.

The $\Upsilon$ distributions for left- and right-handed $\tau$ leptons have different shapes in case of the $\tau ^{\pm }\rightarrow h^{\pm } N \pi ^0\nu$, $N\ge 1$ decay modes, while for the $\tau ^{\pm }\rightarrow h^{\pm } \nu$ mode the polarisation sensitivity comes mostly from different acceptances and efficiencies. The branching ratio of the $\tau ^{\pm }\rightarrow h^{\pm }\pi ^0\nu$ decay mode also exceeds the total branching ratio of the remaining single-prong $\tau _\text {had}$ decay modes combined.

Most of the sensitivity originates from the $\tau ^{\pm }\rightarrow h^{\pm }\pi ^0\nu$ decays. The $\tau ^{\pm }\rightarrow h^{\pm }\nu$ and other remaining modes have similar individual sensitivities and they also make a significant contribution to the overall polarisation sensitivity.

## Background estimate

The signal topology can be mimicked by several background processes, which require different strategies for their estimation. The two largest background contributions arise from multijet and *W*+jets events. In multijet events both the lepton and $\tau _\text {had}$ candidates originate from quark- or gluon-initiated jets. They contribute about 19% (7%) of the total event yield in the $\tau _e\textendash \tau _\text {had}$ ($\tau _\mu \textendash \tau _\text {had}$) channel. In most of the *W*+jets background events, a lepton is produced in the decay of the *W* boson and a jet is misidentified as a $\tau _\text {had}$ lepton. They contribute about 7% (8%) of the events in the $\tau _e\textendash \tau _\text {had}$ ($\tau _\mu \textendash \tau _\text {had}$) channel. Both major backgrounds are estimated using data-driven techniques, which are described in this section. The control regions utilised for these estimates are compiled in Table 3. A minor background contribution consists of $(Z/\gamma ^{*}\rightarrow \ell \ell )$+jets ($\ell =e, \mu$) events, where $\tau _\text {had}$ candidates can originate from quark- or gluon-initiated jets or from one of the leptons. Another background stems from events with top pairs which involve a real lepton and either a real $\tau _\text {had}$ or a quark- or gluon-initiated jet that is misidentified. These minor background contributions are estimated from the simulation. They are normalised with their respective cross-sections and corrections for differences in (mis-) identification between data and the simulation are applied. They amount to about 5% (2%) of the total event yield in the $\tau _e\textendash \tau _\text {had}$ ($\tau _\mu \textendash \tau _\text {had}$) channel.

**Table 3:** Summary of the control regions used for the background estimates

Region	Event selection changes compared to selected signal region
Same-sign region	Inverted opposite-charge-sign requirement
Opposite-sign multijet control region	Inverted lepton-isolation requirement
Same-sign multijet control region	Inverted lepton-isolation and opposite-charge-sign requirement
Opposite-sign *W*+jets control region	$\sum \Delta \phi \ge 3.5$, $m_{\text {T}} > 70$ GeV (instead of $\sum \Delta \phi < 3.5$, $m_{\text {T}} < 30$ GeV)
Same-sign *W*+jets control region	$\sum \Delta \phi \ge 3.5$, $m_{\text {T}} > 70$ GeV (instead of $\sum \Delta \phi < 3.5$, $m_{\text {T}} < 30$ GeV),
	Inverted opposite-charge-sign requirement

### Estimation of *W*+jets background

**Fig. 2:**
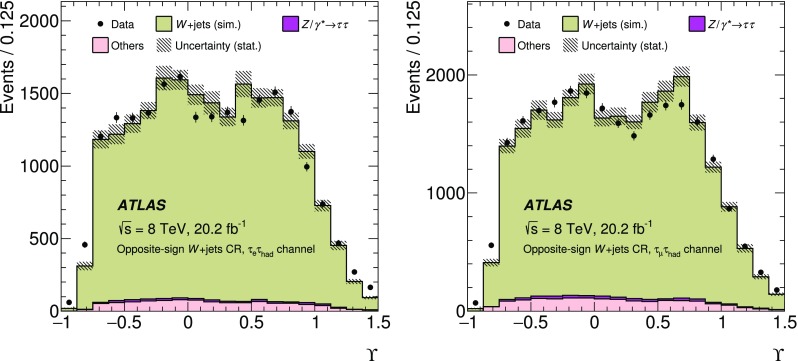
The $\Upsilon$ distribution in the opposite-sign *W*+jets control region in the $\tau _e\textendash \tau _\text {had}$ (left) and $\tau _\mu \textendash \tau _\text {had}$ (right) channel. The contributions of $Z/\gamma ^{*}\rightarrow \tau \tau$ and of $Z/\gamma ^{*}\rightarrow \ell \ell$ and $t\bar{t}$ (other) events are estimated from the simulation. The $\tau$ polarisation in $Z/\gamma ^{*}\rightarrow \tau \tau$ events is obtained from the simulation. The shape of the *W*+jets contribution is estimated from the simulation as well. The *W*+jets contribution is normalised such that the total estimated event yield matches the observed yield. Only statistical uncertainties are shown

The *W*+jets background is estimated from a dedicated control region, which is defined by inverting the $\sum \Delta \phi$ requirement applied in the signal region selection and altering the transverse mass requirement to $m_{\text {T}}>70$ GeV (see Table 3). Figure 2 shows the $\Upsilon$ distribution in the *W*+jets control region with data and simulation overlaid.

Even though the simulation provides a reasonable description of the shape of the $\Upsilon$ distribution in *W*+jets events, a more precise and robust description is utilised. It is obtained from the large number of *W*+jets events in the control region. For this, the $\Upsilon$ distribution for *W*+jets events in the control region is estimated by subtracting the $Z/\gamma ^{*}\rightarrow \ell \ell$, $Z/\gamma ^{*}\rightarrow \tau \tau$ and $t\bar{t}$ contributions as predicted by the simulation from the data. Here, the $\tau$ polarisation in $Z/\gamma ^{*}\rightarrow \tau \tau$ events is taken from the simulation. However, the $W+$jets estimate is only negligibly affected if the $\tau$ polarisation in $Z/\gamma ^{*}\rightarrow \tau \tau$ events is assumed to be $-1$ or $+1$ instead of being taken from the simulation, because the signal contamination in the *W*+jets control region is very small (below 1%). Due to the strict transverse mass requirement, the multijet contribution in the *W*+jets control region is negligible and it is thus ignored.

Possible differences between the $\Upsilon$ distributions in *W*+jets events in the *W*+jets control region and the selected signal region are assessed by performing a linear fit to the ratio of these distributions in simulated *W*+jets events. The fit functions describe the ratios within statistical uncertainties in both channels. The resulting slopes are $0.03\pm 0.05$ ($-0.02\pm 0.05$) in the $\tau _e\textendash \tau _\text {had}$ ($\tau _\mu \textendash \tau _\text {had}$) channel and are used to perform linear corrections when transferring the $W+$jets $\Upsilon$ templates from the $W+$jets control region to the selected signal region.

Additionally, the impact of altering the $\sum \Delta \phi$ and $m_{\mathrm {T}}$ requirements, which are used to define the $W+$jets control region, was studied using dedicated validation regions. Differences between the $\Upsilon$ distributions in the validation regions and the $W+$jets control region are evaluated using additional linear fits. If one of the resulting slopes lies outside the range covered by the statistical uncertainty in the slope estimated previously, the uncertainty is inflated until the difference is covered. This results in an inflation of the slope uncertainty in the $\tau _\mu \textendash \tau _\text {had}$ channel by a factor of 1.2. The slope uncertainty in the $\tau _e\textendash \tau _\text {had}$ channel remains unchanged. The resulting uncertainties are referred to as *W*+jets shape uncertainties.

The normalisation of the *W*+jets contribution in the selected signal region is determined by multiplying the event yield predicted from simulation by the ratio of the *W*+jets event yields observed and predicted in the *W*+jets control region. The ratio is about 0.8 in both channels.

An uncertainty of 3% originates from the limited size of the simulated event samples and is considered as a systematic uncertainty.

### Estimation of multijet background

The multijet background is estimated as follows. The shape of the $\Upsilon$ distribution is estimated from the same-sign control region, in which the opposite-sign requirement on the lepton and $\tau _\text {had}$ candidates is reversed (see Table 3). The ratio $r_\text {QCD}$ of multijet event yields with opposite charge sign and same charge sign is used to scale the distribution obtained in the same-sign region. This ratio is measured in dedicated multijet control regions in which the lepton isolation requirements are inverted.

In order to obtain the multijet contribution in the same-sign and multijet control regions, the contributions from *W*+jets, $Z/\gamma ^{*}\rightarrow \ell \ell$, $Z/\gamma ^{*}\rightarrow \tau \tau$, and $t\bar{t}$ events are subtracted from the data. These contributions amount to about $28\%$ ($45\%$) of the data yield in the same-sign region in the $\tau _e\textendash \tau _\text {had}$ ($\tau _\mu \textendash \tau _\text {had}$) channels and to at most $16\%$ in the multijet control regions. The $Z/\gamma ^{*}\rightarrow \ell \ell$, $Z/\gamma ^{*}\rightarrow \tau \tau$, and $t\bar{t}$ contributions are estimated and the $\tau$ polarisation in $Z/\gamma ^{*}\rightarrow \tau \tau$ events is taken from the simulation. As in the $W+$jets background estimate, an altered polarisation would have a negligible effect on the multijet estimate. The $W+$jets contribution in the same-sign region is estimated in the same way as in the selected signal region using the same-sign *W*+jets control region. The $W+$jets contribution in the opposite-sign (same-sign) multijet control region is estimated as in the signal (same-sign) region.

The value of $r_\text {QCD}$ in the $\tau _e\textendash \tau _\text {had}$ ($\tau _\mu \textendash \tau _\text {had}$) channel is 1.05 (1.12), and the statistical uncertainty is negligible. The systematic uncertainty is estimated by studying the dependence of the ratio of opposite-sign and same-sign event yields on the lepton isolation from well-isolated to not isolated leptons. It is found to be $10\%$ ($9\%$) in the $\tau _e\textendash \tau _\text {had}$ ($\tau _\mu \textendash \tau _\text {had}$) channel.

**Fig. 3:**
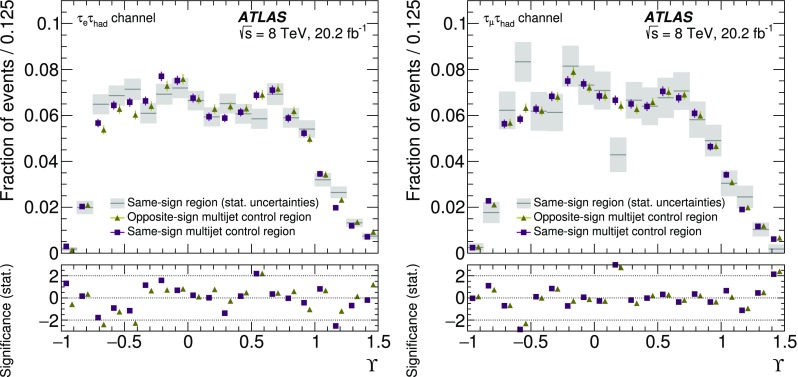
Multijet templates obtained in the same-sign region and in the opposite- and same-sign multijet control regions in the $\tau _e\textendash \tau _\text {had}$ (left) and $\tau _\mu \textendash \tau _\text {had}$ (right) channel. Only statistical uncertainties are shown. The significances, calculated from the statistical uncertainties, of the differences between the shapes in the same-sign region and those in the multijet control regions are shown as well

The multijet background estimate relies on the assumption that the shape of the $\Upsilon$ distribution is the same for multijet events with opposite and same sign lepton and $\tau _\text {had}$ candidates. This is verified by comparing the distributions in the opposite-sign and same-sign multijet control regions and in the same-sign region (see Fig. 3). The shapes agree within the statistical uncertainties in the same-sign region.

## Systematic uncertainties

The extraction of the $\tau$ polarisation from the observed data relies on the prediction of the signal and background $\Upsilon$ templates. Systematic uncertainties can affect the shape of the templates, as well as the acceptance and thus the normalisation. The most important uncertainties are those that can alter the shapes of the signal templates.

**Fig. 4:**
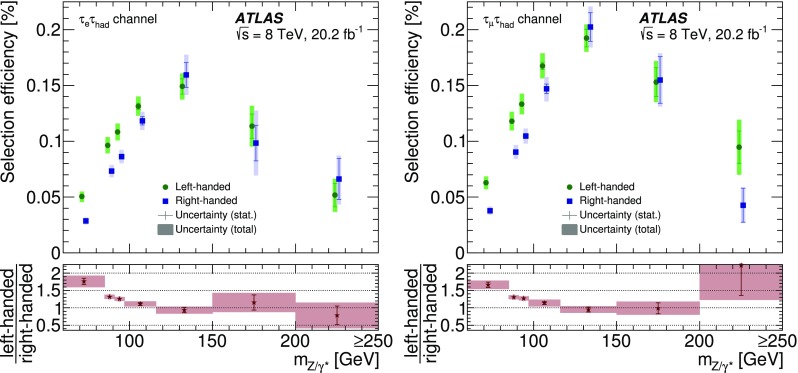
Selection efficiency for signal events in the $\tau _e\textendash \tau _\text {had}$ (left) and $\tau _\mu \textendash \tau _\text {had}$ (right) channels as a function of $m_{Z/\gamma ^{*}}$. No requirement is placed on the $\tau$ decay modes at stable-particle level. The statistical and total uncertainties are indicated. The statistical and total uncertainties in the efficiency ratio are shown. The last bin includes overflow events

Signal acceptance uncertainties affect the left- and right-handed signal components in a very similar way, which means that they have less impact in this analysis. As the background contamination is relatively small (about $30\%$ ($20\%$) in the $\tau _e\textendash \tau _\text {had}$ ($\tau _\mu \textendash \tau _\text {had}$) channel), the systematic uncertainties associated with its estimate have a minor impact on the measurement. The uncertainties are discussed below, grouped into experimental and theoretical uncertainties. Modelling uncertainties for the data-driven background estimates are discussed in Sect. 7. A detailed summary of the event yields expected in the selected signal region with full uncertainties can be found in Table 4. Figure 4 shows the selection efficiency of events with left- and right-handed $\tau ^{-}$ as a function of $m_{Z/\gamma ^{*}}$ for use in the interpretation of this measurement. Signal inefficiencies are dominated by decay mode and kinematic acceptance requirements.

**Table 4:** Event yields expected in the selected signal region for both channels. The $Z/\gamma ^{*}\rightarrow \tau \tau$ contribution is shown separately for the three components used when extracting the polarisation in the 66–116 GeV mass-selected region (see Sect. 5). The $\tau$ polarisation is assumed from the simulation for $Z/\gamma ^{*}\rightarrow \tau \tau$ events. Total uncertainties are shown

Process	$\tau _e\textendash \tau _\text {had}$ channel	$\tau _\mu \textendash \tau _\text {had}$ channel
Data	32,243	32,347
Total expected	32,000$\,^{+1600}_{-1600}$	32,800$\,^{+1800}_{-1800}$
Left-handed	13,800$\,^{+1100}_{-1100}$	17,000$\,^{+1400}_{-1300}$
Right-handed	7800$\,^{+600}_{-600}$	9600$\,^{+700}_{-700}$
Outside mass-selected region	430$\,^{+40}_{-40}$	550$\,^{+40}_{-40}$
$W+$jets	2240$\,^{+260}_{-240}$	2590$\,^{+210}_{-220}$
Multijet	6200$\,^{+600}_{-600}$	2370$\,^{+270}_{-300}$
Top pair	360$\,^{+40}_{-40}$	390$\,^{+40}_{-40}$
$(Z/\gamma ^{*}\rightarrow \ell \ell )+$jets	1210$\,^{+140}_{-140}$	360$\,^{+50}_{-40}$

### Experimental uncertainties

Experimental sources of uncertainty include trigger, object reconstruction and identification efficiencies, energy and momentum scales and resolutions, and the measurement of the integrated luminosity. They are described below in the order of importance.

The efficiency for identifying $\tau _\text {had}$ candidates was measured in data using tag-and-probe techniques and is about 55% for single-prong $\tau$ leptons for the ‘medium’ working point used in this analysis [40]. The relative uncertainty in the $\tau _\text {had}$ identification efficiency is $(2\textendash 3)\%$ for single-prong $\tau$ candidates. The simulated event samples are corrected for differences in the overall efficiency between data and simulation and the associated uncertainties in the normalisation of the signal and background templates are propagated through the analysis. Some of the input variables [40] used in $\tau _\text {had}$ identification are strongly correlated with $\Upsilon$. A mismodelling of these input variables may thus cause differences between the shapes of the $\Upsilon$ distributions in data and the simulation causing errors specific to this analysis. These errors were studied in detail and are estimated by comparing the $\tau _\text {had}$ identification input variable distributions of $\tau _\text {had}$ candidates in *W*+jets and top pair events in the data and the simulation. The observed differences are propagated through the analysis. The resulting uncertainties are referred to as $\tau _\text {had}$ identification uncertainties in the following.

The modelling of $$\Upsilon $$ strongly relies on the modelling of the energy response to $$\tau _\text {had} $$, because the reconstructed $$\tau $$ energy is a direct input (see Eq. 3). In contrast to observables such as masses of heavy particles, which are commonly exploited in analyses studying decay channels that involve $$\tau $$ leptons, the reconstruction of $$\Upsilon $$ is unaffected by the presence of neutrinos in $$\tau $$ decays. It is therefore particularly sensitive to the modelling of the $$\tau _{\text {had}-\text {vis}} $$ energy response. Consequently, detailed studies were performed to provide a thorough understanding of the related uncertainties. The Tau Energy Scale (TES) uncertainty for $$\tau _\text {had} $$ decays is evaluated based on the single-hadron response in the calorimeters that was studied in Ref. [40]. The uncertainty is a function of $$\eta $$ and $$E_ T $$ and is generally near $$3\%$$. A mismodelling of the energy response to hadrons and to photons may affect the $$\Upsilon $$ templates in different ways. For $$\tau _\text {had} $$ candidates with $$\Upsilon $$ values around $$+1$$, most of the energy originates from hadrons, mostly charged pions. Conversely, photons that arise from $$\pi ^0$$ decays typically carry a large fraction of the energy for candidates with $$\Upsilon $$ values close to $$-1$$. This is accounted for by splitting the TES uncertainty from Ref. [40] into hadronic and electromagnetic components based on the stable-particle level fraction of the $$\tau _{\text {had}-\text {vis}} $$ energy carried by hadrons and photons, respectively, for signal events.

A mismodelling of the $\tau _\text {had}$ energy resolution (TER) may affect the modelling of the $\Upsilon$ distribution as well and may be distinguishable from the effect caused by a mismodelling of the TES. The TER in ATLAS was not measured before and is therefore evaluated in this analysis. The TER uncertainties are considered for the hadronic and electromagnetic components separately and determined from the $\Upsilon$ distribution in the same fit in which the polarisation is measured. The absolute uncertainties are found to be 1.4% for the hadronic and 1.8% for the electromagnetic TER component.

The TES and TER uncertainties are each considered separately for the hadronic and electromagnetic components. The TES uncertainty from the single-hadron response studies is also considered for the backgrounds, which are estimated from the simulation. Here, the contribution from $Z/\gamma ^{*}\rightarrow ee$ events, for which the selected $\tau _\text {had}$ candidate originates from an electron, is treated separately from the remaining backgrounds, for which the $\tau _\text {had}$ candidates originate from quark- or gluon-initiated jets.

The remaining experimental uncertainties, referred to as other uncertainties, have a minor effect on the final result:

- Trigger, reconstruction and identification of electrons and muons: The efficiencies for triggering, reconstructing, and identifying electrons and muons are measured in data using tag-and-probe techniques. Electron energy and muon momentum corrections and their uncertainties are evaluated by comparing the response in data and in the simulation [34, 35]. The simulated event samples are corrected for the differences.
- Tag-and-probe studies of $Z/\gamma ^{*}\rightarrow ee$ events are used to derive the correction factors on the rate of electrons to be misidentified as $\tau _\text {had}$ leptons, as well their uncertainties [40].
- Uncertainties that affect the $$E_ T ^ miss $$ estimation: In this analysis, uncertainties in the jet energy scale (JES) and resolution (JER) are only relevant due to their effect on the $$E_ T ^ miss $$ reconstruction. Various sources of JES and JER uncertainty are considered [44]. Along with the TES, TER, electron energy, and muon momentum uncertainties, they are propagated to the $$E_ T ^ miss $$ calculation. Additional uncertainties in the $$E_ T ^ miss $$ scale and resolution due to energy clusters that do not belong to any reconstructed object are considered as well [41].
- Luminosity: The absolute luminosity scale is derived from beam-separation scans performed in November 2012. The uncertainty in the integrated luminosity is $1.9\%$ [45]. It applies to simulated samples.

The uncertainties described above are propagated through the analysis.

### Theory uncertainties

Theory uncertainties in the signal templates include uncertainties in the event-by-event calculation of the helicity in the signal sample using the TauSpinner algorithm, the choice of signal event generator and its parton shower simulation model, and the choice of PDFs.

The uncertainty related to the signal sample splitting with the TauSpinner algorithm is estimated by varying the relevant TauSpinner input parameters. These are the QCD factorisation and renormalisation scales, the $\alpha _\text {s}$ coupling and the PDFs. Since the uncertainties may be mass dependent, they are calculated for three different mass ranges around the *Z* boson peak (66–116, 81–101, and 88–92 GeV). One of them coincides with the 66 $< m_{Z/\gamma ^{*}}<$ 116 GeV range used in this analysis. Samples of $pp \rightarrow \tau \tau +2$ jets events generated with the MadGraph [46] event generator interfaced with the Pythia8 [21] hadronisation and $\tau$ decay modelling and the same methods as in Ref. [18] are used. The signal samples used in the analysis were generated with different $\sin ^2\theta _\text {W}^\text {eff}$ values set in the Alpgen event generator and Pythia6+Tauola hadronisation and $\tau$ decay modelling. This may result in an additional uncertainty in the sample splitting. To assess this uncertainty, the polarisation obtained via the method described in Sect. 5 is compared to the polarisation reported by the TauSpinner algorithm. The difference is considered as a systematic uncertainty. The two sources of signal sample splitting uncertainty have a similar impact. Based on these studies, the signal template variations that are caused by 1% migrations from the left-handed to right-handed signal subsamples and vice versa are considered and propagated through the analysis. The resulting uncertainties are referred to as signal sample splitting uncertainties.

The uncertainty related to the choice of event generator for the signal sample is estimated with the help of two auxiliary samples produced with the Pythia8 event generator and with the Powheg event generator interfaced with the Pythia8 hadronisation and $\tau$ decay modelling (see Table 1). Because the latter was generated using the CT10 PDF set, it is reweighted to match the default one (CTEQ6L1) with the LHAPDF package [47] to avoid double-counting of possible systematic effects. These two samples are used to obtain a set of event weights relative to the default Alpgen sample before any event selection with respect to the kinematics of $\tau$ leptons and *Z* bosons and to the $\Upsilon$ spectra of various hadronic $\tau$ decay modes.

The resulting uncertainties are among the leading ones in the analysis. Most of the impact arises from the uncertainties in the $\tau$ lepton pseudorapidity distributions and from the uncertainties in the $\Upsilon$ distributions in $\tau ^{\pm }\rightarrow h^{\pm }\pi ^{0}\nu$ decays. The estimation of the uncertainties related to the event generator in the measurement of the polarisation in the fiducial region is performed in the same way as described above. The uncertainties are referred to as signal modelling uncertainties.

The parton shower simulation model uncertainty is estimated using an auxiliary signal sample produced with the Alpgen event generator interfaced the Herwig hadronisation modelling instead of the Pythia6 hadronisation modelling as in the default sample. It is used to obtain a set of event weights relative to the default Alpgen sample before any event selection in the same way as for the uncertainties related to the event generator choice described above.

The impact of this systematic uncertainty, which is included in the other uncertainties category, on the final result is negligible.

The PDF-induced uncertainty is estimated by performing a reweighting of the signal sample using the LHAPDF package. The nominal PDF set CTEQ6L1 is reweighted to the following alternative LO PDF sets: NNPDF30$\_$LO$\_$AS$\_$0118, MMHT2014LO68CL, and CT14LO. The uncertainties are estimated for all three alternative PDF sets and found to be largest for the CT14LO PDF set. The contribution of PDF uncertainties to the final polarisation uncertainty is small.

## Fit model

The $\tau$ polarisation is extracted in an extended, binned maximum-likelihood fit to the $\Upsilon$ distribution. The probability density function is constructed in the histogram-based fitting tool HistFactory [48] within the RooFit framework [49]. The fit is performed simultaneously in the signal and same-sign regions, each with 20 equally spaced bins in the range $[-1,1.5]$ in $\Upsilon$, in both the $\tau _e\textendash \tau _\text {had}$ and $\tau _\mu \textendash \tau _\text {had}$ channels. The fit to the observed data distribution is performed twice, first to extract the $\tau$ polarisation in the range of $66<m_{Z/\gamma ^{*}}<116\hbox { GeV}$ and then to measure the polarisation in the fiducial region.

The signal histograms of the $\Upsilon$ variable for the fit that extracts the polarisation in the mass-selected region are the respective three $Z/\gamma ^{*}\rightarrow \tau \tau$ contributions (see Sect. 5) that pass the selected signal region and same-sign region event selections in the simulation. They are passed to the fit as nominal signal templates. The left-handed and right-handed signal templates describing events inside the mass-selected region are each normalised to the full $Z/\gamma ^{*}\rightarrow \tau \tau$ cross-section inside the mass-selected region. The relative contributions are scaled with the parameter of interest, $P^\text {POI}_{\tau }$, such that $P^\text {POI}_{\tau }$ represents the polarisation at production as defined in Eq. (1) without any selection except the $66<m_{Z/\gamma ^{*}}<116\hbox { GeV}$ requirement. The template for $Z/\gamma ^{*}\rightarrow \tau \tau$ events outside the mass-selected region is scaled with the respective $Z/\gamma ^{*}\rightarrow \tau \tau$ cross-section and is not affected by the parameter $P^\text {POI}_{\tau }$. Effects causing deviations of the expected polarisation from that in the data could also alter the $Z/\gamma ^{*}\rightarrow \tau \tau$ normalisation. Hence an additional unconstrained fit parameter, $\alpha _Z$, is included to scale the overall normalisation of the $Z/\gamma ^{*}\rightarrow \tau \tau$ signals. The $P^\text {POI}_{\tau }$ and $\alpha _Z$ parameters are common to the fitted relative and overall normalisation of the signal templates in all regions.

The signal templates used in the fit that extracts the $\tau$ polarisation in the fiducial region are obtained in a similar way using the respective three contributions defined in Sect. 5. Here, the left- and right-handed signal templates corresponding to events inside the fiducial region are each scaled with the full $Z/\gamma ^{*}\rightarrow \tau \tau$ cross-section inside the fiducial region. Due to this scaling $P^\text {POI}_{\tau }$ then represents the polarisation of $\tau$ leptons produced in the fiducial region. The contribution made by events outside the fiducial region is treated as previously described for the events outside the mass-selected region. The scaling with $P^\text {POI}_{\tau }$ and $\alpha _Z$ is also done as described for the mass-selected region. The treatment of the backgrounds and systematic uncertainties is described below.

The *Z*+jets and $t\bar{t}$ backgrounds are taken into account by the simulated $\Upsilon$ distributions passing the selected signal region and same-sign region event selections. The *W*+jets template histograms are taken from the data-driven estimate. Each of the *Z*+jets, *W*+jets, and $t\bar{t}$ background templates are normalised to the expected number of events for each background in the respective regions as described in Sect. 7. The multijet background is estimated in a simultaneous fit in the signal and same-sign regions with nuisance parameters common to the two regions per bin and channel to fit the content in each. The related uncertainties are referred to as multijet estimate uncertainties. For each channel the normalisation of the multijet background in the selected signal region relative to the same-sign region is scaled via a fixed normalisation parameter, $r_\text {QCD}$.

A summary of the nuisance parameters related to systematic uncertainties can be found in Table 5. All systematic uncertainties in the same-sign region are much smaller than the statistical uncertainties in the multijet estimate. They are thus negligible and omitted in the fit.

**Table 5:** Summary of nuisance parameters related to systematic uncertainties considered in the fits that extract the $\tau$ polarisation when combining the two channels. The number of parameters in the ‘Other’ category is 36 (34) in the fit that extracts the polarisation in the mass-selected region (in the fiducial region)

Source of uncertainty	Number of parameters	Constraint	Steer variation of
Multijet estimate	40	None	One bin each
MC statistical	40	Poissonian	One bin each
Modelling of signal process	3	Gaussian	Shape and normalisation
$\tau _\text {had}$ identification	5	Gaussian	Shape or normalisation
Signal sample splitting	2	Gaussian	Shape and normalisation
TES and TER	6	Gaussian	Shape and normalisation
PDF	1	Gaussian	Shape and normalisation
*W*+jets shape	2	Gaussian	Shape
Other	34 or 36	Gaussian	Normalisation

The statistical uncertainty associated with the finite size of the simulated event samples is accounted for with a variation of the Barlow–Beeston treatment [50]. This results in one nuisance parameter per channel and bin. The related uncertainties are referred to as MC statistical uncertainties.

Further nuisance parameters are included to account for systematic variations of the template shape and normalisation estimated with the methods described in Sects. 7 and 8. The systematic uncertainties are accounted for in the fit with variations of the individual nominal template histograms. These variations may change the overall normalisation of the histogram or may introduce bin-dependent shape differences. In either case, a single nuisance parameter interpolates between variations that correspond to the estimated $+1\sigma$ and $-1\sigma$ uncertainties with a Gaussian constraint. The nuisance parameters may be correlated between normalisation and shape variations, between samples, regions, and channels.

The signal process modelling and PDF parameters control the variations introduced when changing the event generator or the PDF set, respectively. Three of the parameters related to $\tau _\text {had}$ identification uncertainties account for the systematic variation of the input variables that may significantly affect the modelling of the signal template shapes in the simulation. The remaining two $\tau _\text {had}$ identification parameters exclusively vary the normalisation of the signal and background templates according to the uncertainties estimated in the tag-and-probe studies from Ref. [40]. The correlations of the normalisation and shape uncertainties are not known and the parameters are treated as uncorrelated. It was verified that the correlation assumed has a negligible effect on the overall uncertainty. One parameter controls each of the variations caused by migrations from the left-handed to right-handed signal subsamples and vice versa, accounting for the signal sample splitting uncertainties. The correlations of these parameters are also unknown. They are treated as uncorrelated. Their impact on the polarisation uncertainty would be reduced, if they were assumed to be fully correlated instead. One parameter controls each of the variations of the hadronic and electromagnetic components of the TES and TER in the signal templates. The remaining TES parameters account for the TES uncertainty in the backgrounds. One of them is dedicated to $Z/\gamma ^{*}\rightarrow ee$ events, in which one of the electrons is misidentified as a $\tau _\text {had}$ candidate. One *W*+jets shape parameter per channel accounts for the shape uncertainties described in Sect. 7.1.

The remaining systematic uncertainties are considered for their impact on the normalisation of each of the template histograms.

Most of them have a small impact on the templates, individually. In the signal region for each sample, the systematic uncertainties are ordered by decreasing amount of normalisation variation that they cause. Nuisance parameters are included until at least $95\%$ of the sum of all normalisation uncertainties per sample is covered. It was verified that the remaining uncertainties would have a negligible impact, if considered.

The fit model was validated in detail using pseudo-experiments. It was verified that it correctly determines the polarisation when confronted with data samples that include polarisation values different from those found in the simulation. The bias was found negligible and the uncertainties determined by the fit were found accurate.

## Results

**Fig. 5:**
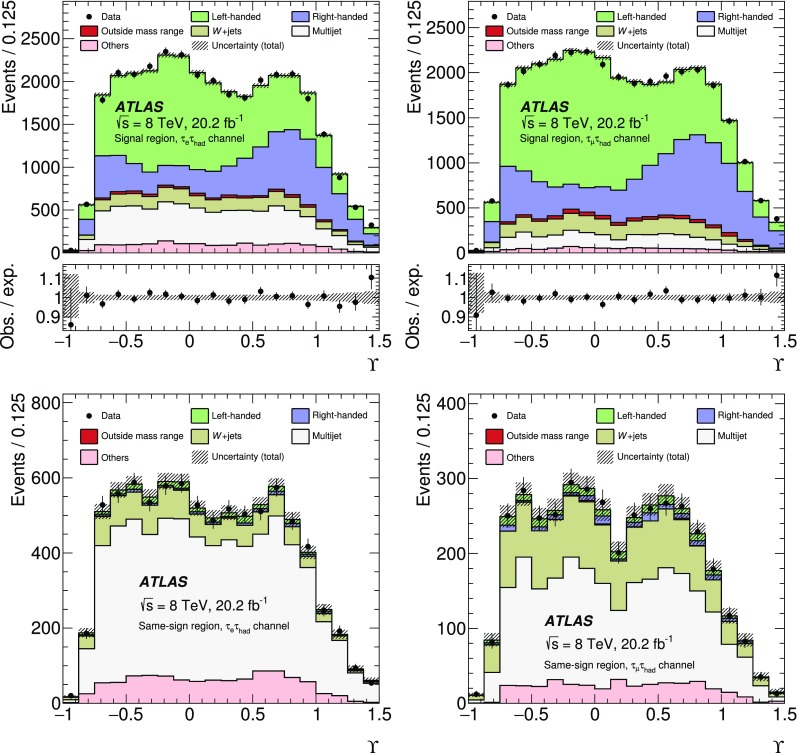
Post-fit $\Upsilon$ distributions for the $\tau _e\textendash \tau _\text {had}$ (left) and $\tau _\mu \textendash \tau _\text {had}$ (right) channels, and for the signal (top) and same-sign (bottom) regions for the fit that extracts the $\tau$ polarisation in the mass-selected region of $66<m_{Z/\gamma ^{*}}<116\hbox { GeV}$

**Fig. 6:**
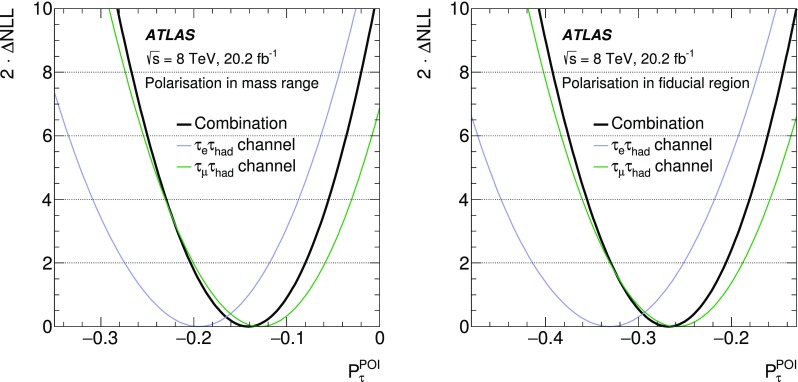
Likelihood profiles of $P^\text {POI}_{\tau }$ for the fits that extract the polarisation in the mass-selected region of $66<m_{Z/\gamma ^{*}}<116\hbox { GeV}$ (left) and in the fiducial region (right). The profiles are shown separately for the fits in the $\tau _e\textendash \tau _\text {had}$ and $\tau _\mu \textendash \tau _\text {had}$ channels and for the combination

The $\tau$ polarisations in the mass-selected region of $66<m_{Z/\gamma ^{*}}<116\hbox { GeV}$, and in the fiducial region, are extracted using the extended, binned maximum-likelihood fit described in Sect. 9. The fit is performed for the individual channels and for the combination. The $\Upsilon$ distributions after the combined fit that extracts the $\tau$ polarisation in the mass-selected region are shown in Fig. 5. The $P^\text {POI}_{\tau }$ likelihood profiles are shown in Fig. 6 and the resulting polarisation values are summarised in Table 6. The polarisation values measured in the $\tau _e\textendash \tau _\text {had}$ and $\tau _\mu \textendash \tau _\text {had}$ channels agree at a level of 1.4 standard deviations and are compatible. Only uncertainties that are uncorrelated between the channels are considered in this compatibility estimate. Apart from the statistical uncertainties, these are the uncertainties related to the finite size of the simulated event samples and those related to the multijet background estimate. Some of the nuisance parameters, which correspond to uncertainties that are specific to this analysis such as the uncertainties in the modelling of $\tau _\text {had}$ identification and $\tau _\text {had}$ energy reconstruction on the $\Upsilon$ distribution, are fit to values that differ from their nominal estimates. The sizes of these ‘pulls’ are similar in the two channels. The largest effect is that the polarisation value obtained in the combination is higher and close to that measured in the $\tau _\mu \textendash \tau _\text {had}$ channel.

The impact of the different sources of uncertainty is summarised in Table 7.

The uncertainty in a $\sin ^2\theta _\text {W}^\text {eff}$ value extracted from this measurement would be approximately 15 times larger than that reached by the LEP experiments from $\tau$ polarisation [1]. Therefore, and because additional studies would be required to correct for the *Z* boson and photon interference, $\sin ^2\theta _\text {W}^\text {eff}$ is not determined here.

**Table 6:** Measured $\tau$ polarisation values and overall uncertainties in the mass-selected region of $66<m_{Z/\gamma ^{*}}<116\hbox { GeV}$ and in the fiducial region

Channel	$P_\tau$ in mass-selected region	$P_\tau$ in fiducial region
$\tau _e\textendash \tau _\text {had}$	$-0.20\pm 0.02\;(\text {stat})\pm \;0.05\;(\text {syst})$	$-0.33\pm 0.03\;(\text {stat})\pm \;0.05\;(\text {syst})$
$\tau _\mu \textendash \tau _\text {had}$	$-0.13\pm 0.02\;(\text {stat})\pm \;0.05\;(\text {syst})$	$-0.26\pm 0.02\;(\text {stat})\pm \;0.05\;(\text {syst})$
Combination	$-0.14\pm 0.02\;(\text {stat})\pm \;0.04\;(\text {syst})$	$-0.27\pm 0.02\;(\text {stat})\pm \;0.04\;(\text {syst})$

**Table 7:** Impact of the individual sources of uncertainty on the polarisation uncertainty $\sigma _{P_\tau }$ in the combined fits that extract the $\tau$ polarisation in the mass-selected region of $66<m_{Z/\gamma ^{*}}<116\hbox { GeV}$ and in the fiducial region. The total systematic uncertainty quoted is estimated from the total uncertainty and the statistical uncertainty

Source of uncertainty	$\sigma _{P_\tau }$ in mass-selected region	$\sigma _{P_\tau }$ in fiducial region
Modelling of signal process	0.026	0.022
$\tau _\text {had}$ identification	0.020	0.024
MC statistical	0.016	0.019
Signal sample splitting	0.015	0.015
TES and TER	0.015	0.019
Multijet estimate	0.013	0.013
PDF	0.007	0.005
*W*+jets shape	0.002	0.003
Other	0.008	0.003
Total systematic uncertainty	0.040	0.039
Statistical uncertainty	0.015	0.016

## Conclusion

A measurement of the $\tau$ polarisation in $Z/\gamma ^{*}\rightarrow \tau \tau$ decays with one leptonic and one single-prong hadronic $\tau$ decay is performed. Sensitivity to $\tau$ polarisation is gained from the hadronic $\tau$ decay. The 20.2 $\text{ fb }^{-1}$ dataset of proton–proton collisions at $\sqrt{s}=8$ TeV collected by the ATLAS experiment at the LHC in 2012 is utilised. The measurement is complementary to previous measurements in electron–positron collisions.

In the fiducial region, the measured $\tau$ polarisation is $P_\tau ~=-0.27\;\pm ~0.02\;(\text {stat})\;\pm ~0.04\;(\text {syst})$. It agrees with the value predicted by the Standard Model (as implemented in the Alpgen event generator interfaced with the Pythia6 and Tauola) hadronisation and $\tau$ decay modelling, which is $P_\tau ~=-0.270\;\pm \;0.006$. The polarisation is then extracted in the mass-selected region of 66 $< m_{Z/\gamma ^{*}}<$ 116 GeV and a value of $P_\tau ~=-0.14\;\pm ~0.02\;(\text {stat})\;\pm ~0.04\; (\text {syst})$ is found. The result is in agreement with Standard Model prediction of $P_\tau ~=-0.1517\;\pm \;0.0019$.

## Footnotes

1 The $\tau$ helicity states are experimentally accessible, in contrast to chiral states, as the kinematic distributions of the $\tau$ decay products are sensitive to the spin of the $\tau$ lepton. The inaccuracies resulting from the assumption of helicity–chirality equivalence are negligible for the $\tau$ decays studied in this analysis.

2 ATLAS uses a right-handed coordinate system with its origin at the nominal interaction point (IP) in the centre of the detector and the *z*-axis along the beam pipe. The *x*-axis points from the IP to the centre of the LHC ring, and the *y*-axis points upwards. Cylindrical coordinates $(r,\phi )$ are used in the transverse plane, $\phi \in (-\pi ,\pi ]$ being the azimuthal angle around the *z*-axis. The pseudorapidity is defined in terms of the polar angle $\theta$ as $\eta = -\ln \tan (\theta /2)$. Angular distance is defined as $\Delta R \equiv \sqrt{(\Delta \eta )^{2} + (\Delta \phi )^{2}}$. The $\Delta \phi$ separation is defined as $\mathrm {min}(|\phi _1-\phi _2|,2\pi -|\phi _1-\phi _2|)$.
